# Decision-ready evidence for vital pulp therapy: a network meta-analysis of bioactive materials in mature permanent teeth

**DOI:** 10.3389/fdmed.2026.1780755

**Published:** 2026-04-20

**Authors:** Firas Elmsmari, Reem B. Abdelsayed, Qamar Albasoumi, Tareq Aljafarawi, Swadheena Patro, Ajinkya M. Pawar

**Affiliations:** 1 Department of Clinical Sciences, College of Dentistry, Ajman University, Ajman, United Arab Emirates; 2 Center of Medical and Bio Allied Health Sciences Research, Ajman University, Ajman, United Arab Emirates; 3 Department of Conservative Dentistry and Endodontics, Kalinga Institute of Dental Sciences, KIIT University, Bhubaneswar, India; 4 Department of Conservative Dentistry and Endodontics, Nair Hospital Dental College, Mumbai, India

**Keywords:** calcium-silicate cements, dental pulp diseases, mineral trioxide aggregate, permanent teeth, pulpotomy, vital pulp therapy

## Abstract

**Objectives:**

This network meta-analysis aimed to generate clinically credible, decision-ready evidence on the comparative effectiveness of bioactive materials used in vital pulp therapy (VPT) of mature permanent teeth with carious exposures, including symptomatic presentations.

**Materials and methods:**

Following PRISMA 2020 and PRISMA-NMA standards (PROSPERO: CRD420251074110), randomized controlled trials evaluating full pulpotomy (FP), partial pulpotomy (PP), direct pulp capping (DPC), and indirect pulp capping (IPC) were systematically identified across six databases. The primary outcome was composite success (clinical and radiographic) at 6, 12, 24, and 36 months. Arm-based Bayesian random-effects network meta-analyses and frequentist sensitivity models (REML–Hartung–Knapp) were performed. Certainty of evidence was graded using CINeMA.

**Results:**

The primary meta-analysis included a total of thirty-five RCTs with a total of 2906 teeth, of which a total of twenty-four RCTs involving mature/ permanent teeth were included. Success rates across all VPT types and time points of the 24 RCTs were generally comparable at >93%. Hydraulic calcium silicate materials (MTA & Biodentine) showed a greater likelihood of success than Ca(OH)2 in direct pulp caps and partial pulpotomy procedures. The difference was established with high-certainty evidence for the comparison of MTA vs. Ca(OH)2 and moderate-certainty evidence for the comparison of Biodentine vs. Ca(OH)2. In contrast, the comparisons of calcium silicate materials were frequently overlapped in their credible intervals with moderate certainty evidence; thus, no explicit difference was found between the calcium silicate materials (e.g., MTA & Biodentine). If adjunctive therapies (e.g., platelet-rich plasma & laser therapies) have a promising effect on success rates, they were supported with low-certainty evidence based on limited data and network sparsity. The meta-analysis met the requirement for network coherence (*p* > 0.10) and the amount of heterogeneity found was low (*τ*^2^ = 0.09).

**Conclusion:**

Vital pulp therapy in mature permanent teeth demonstrates consistently high success rates, particularly when using calcium silicate-based materials. These findings are supported by high-certainty evidence for comparisons with calcium hydroxide. However, differences among calcium silicate materials remain uncertain due to overlapping credible intervals and moderate certainty of evidence, while adjunctive therapies are supported by low-certainty evidence and should be interpreted cautiously.

**Systematic Review Registration:**

https://www.crd.york.ac.uk/PROSPERO/view/CRD420251074110, PROSPERO CRD420251074110.

## 1 Introduction

Preserving the vitality of dental pulp after carious exposure has become a transformative goal in restorative dentistry and endodontics, moving beyond the traditional approach of root canal therapy (RCT) and raising critical questions as to the best treatment protocols for permanent teeth with pulpal compromise (1). In mature permanent teeth that would once have been routed almost automatically to nonsurgical root canal treatment, vital pulp therapy has re-emerged as a credible first-line option when diagnosis, asepsis, hemostasis, biomaterial selection, and immediate coronal sealing are executed with rigor (2). The American Association of Endodontists’ position statement codified this shift by detailing diagnostic criteria, advocating sodium hypochlorite for hemostasis, and emphasizing the importance of a well-sealed definitive restoration placed without delay (3). It also acknowledges that teeth previously labeled as having irreversible pulpitis may still heal if the inflamed coronal tissue is judiciously removed and the remaining tissue is protected with an appropriate bioactive material (4).

The biological rationale is compelling. The dentine–pulp complex is a vascularized, innervated tissue that contributes to defense, repair, and regulated dentinogenesis (5). Retaining vitality preserves proprioception and immune surveillance, capacities that are unavoidably lost following root canal treatment (6). Recent experimental and translational data supports the resilience of inflamed pulp tissue. Preclinical studies have shown that short-term preoperative inflammation does not negatively affect radiographic or histologic outcomes after complete pulpotomy (7, 8). Therefore, removal of an inflamed coronal pulp does not negatively affect the regenerative capacity of the remaining tissue (9, 10). These results provide additional biological support for extending the use of vital pulp therapy to those teeth diagnosed with non-symptomatic irreversible pulpitis when proper case selection and aseptic techniques are utilized. Contemporary clinical research has begun to validate these advantages under symptomatic conditions in adults (11). Randomized evidence comparing full pulpotomy with root canal therapy in mature molars diagnosed with irreversible pulpitis indicates similar clinical success together with shorter procedure time and lower costs for pulpotomy, strengthening the case that vital pulp therapy is not a compromise but a principled option in many adult cases (12–14).

Advances in materials science have driven much of this change. Calcium hydroxide, the historical standard for pulp capping, is limited by solubility, a tendency toward interfacial leakage, and the formation of porous, brittle barriers (15). Hydraulic calcium-silicate cements such as mineral trioxide aggregate and Biodentine provide bioactivity, superior sealing, and more homogeneous hard-tissue bridges (16). Recent syntheses converge on two findings that matter at the chairside. First, compared with calcium hydroxide, modern silicate cements achieve higher success rates for direct pulp capping and pulpotomy in mature permanent teeth. Second, head-to-head differences among the leading silicates are often small or imprecisely estimated, especially once data are stratified by modality and follow-up window (17, 18). A recent network meta-analysis based on randomized trials found calcium hydroxide consistently underperformed over common follow-ups, while mineral trioxide aggregate remained a high-performing comparator and Biodentine showed no clear inferiority, although precision varied with network density (19). Recent focused reviews on direct pulp capping also report superior performance of mineral trioxide aggregate over calcium hydroxide, with Biodentine performing similarly to mineral trioxide aggregate in many datasets (20, 21).

In terms of the mechanics behind how they work, the effectiveness of modern biomaterials in the clinic is closely related to the chemistry and biology of the materials that they are made from. For example, hydraulic calcium-silicate cements, which can, among other things, provide a source of both calcium ions and hydroxyl ions, create a more alkaline environment conducive to establishing antimicrobial effects as well as promote the mineralization of the cement material through the formation of hydroxyapatite-like materials (22). In addition, the chemical interaction of these materials with dentinal fluids results in the formation of a mineralized layer at the interface between the cement and the tooth/dentin surface, which can improve the sealing ability of the cement and provide long-term durability (23). Also, the recent advances in biomaterials research have provided more support for the role of nanostructured and bioengineered components in enhancing antibacterial properties, kinetics of ion release, and biological response by cells, which are indicative of a larger trend toward biologically responsive material designs in endodontic treatment. These advances indicate that, while sealing effectiveness is important, the ability of the material to alter the biological environment and encourage regeneration of the surrounding tissue is equally important (24).

The decision to use vital pulp therapy in an adult with pain, however, is not made in the abstract. Three clinical axes shape prognosis and therefore must shape evidence synthesis. First is the diagnostic frame. In symptomatic irreversible pulpitis, inflammation is spatially heterogeneous. Coronal tissue may be irreversibly inflamed while radicular tissue retains healing capacity when microbial load is reduced and an effective barrier is established. High-quality reviews focused on mature teeth with this diagnosis support the biological plausibility and clinical viability of vital pulp therapy but call for stronger modality-specific comparisons and longer follow-up. Second is apex maturity. Immature apices present a more permissive healing biology yet differ from mature teeth in periapical environment and occlusal function, so conclusions drawn from young permanent teeth cannot simply be transferred to adult cases. Third is the restoration interface. Over time the integrity of the coronal seal may amplify or erase any advantage conferred by a premium bioceramic (25–27). Outcomes research in endodontics repeatedly identifies restoration quality as a predictor of healing and long-term survival, which means that material choice and restorative execution form a single biological system rather than separable steps (27).

Methodologically, the evidence base has matured but remains heterogeneous in ways that matter for translation into practice. Conventional pairwise meta-analyses often pool across distinct vital-pulp-therapy modalities, which risks masking procedure-specific signals (19, 28). Clinically important moderators such as preoperative diagnosis, tooth type, apex status, and hemostasis protocol are inconsistently reported and rarely modelled (29). Time windows vary across trials, and durability beyond the first year is less frequently documented (30). Network meta-analyses help by integrating direct and indirect evidence; nonetheless, many syntheses present treatment ranks without an explicit grading of the certainty of key contrasts using a framework tailored to networks (31). Without such grading, clinicians are left with an ordering but insufficient insight into transitivity, imprecision, heterogeneity, and incoherence, all of which bear directly on the credibility of a putative hierarchy.

Within this clinical and methodological context, there remains real uncertainty about the comparative effectiveness and practical ranking of the most frequently used materials across the four principal vital-pulp-therapy modalities in adults, especially in symptomatic cases (32). Randomized trials often report high short-term success yet show divergence at longer follow-up or under different restorative protocols. Failures attributable to diagnosis error, technique sensitivity, inadequate isolation, or intrinsic material limitations continue to occur. The literature also makes clear that restorative workflow is not a footnote (33). Immediate, well-sealed definitive restorations and rigorous rubber-dam isolation are central to the biological logic of vital pulp therapy, since bacterial control is the currency that allows the pulp to capitalize on the regenerative potential of hydraulic calcium-silicate cements (34).

The present study addresses these issues by synthesizing randomized evidence across the four modalities in mature permanent teeth with carious exposures, including symptomatic presentations. We harmonize success as a composite of clinical and radiographic outcomes at clinically meaningful time points, model prespecified moderators with *a priori* biological plausibility, report both relative and absolute effects with prediction intervals, and grade the confidence of decision-critical contrasts using an approach explicitly developed for networks. In doing so, we aim to generate hierarchies that are clinically credible rather than merely numerical, and to frame the practical trade-offs that clinicians face when choosing both a procedure and a material for an individual adult tooth.

Against this background, the aim of this study is to generate clinically credible, decision-ready evidence on the comparative effectiveness of bioactive materials used in vital pulp therapy (VPT) for mature permanent teeth with carious exposures, including symptomatic presentations. Objectives: (1) to synthesize randomized evidence across indirect/direct pulp capping, partial pulpotomy, and full pulpotomy using a standardized composite success outcome (clinical + radiographic) at 12 and 24 months; (2) to estimate both relative and absolute effects with prediction intervals and grade confidence using a network-appropriate framework; and (3) to evaluate whether material performance varies by modality after modeling prespecified moderators—diagnosis (with emphasis on symptomatic irreversible pulpitis), apex maturity, tooth type, hemostasis protocol—and explicitly accounting for the restorative interface as part of the biological system.

## 2 Methodology

### 2.1 Protocol, reporting, and registration

This review followed PRISMA 2020 and PRISMA-NMA guidance and was prospectively registered in PROSPERO (CRD420251074110). All eligibility criteria, endpoints, moderators, and primary analysis plans were specified *a priori*; any deviations are reported with justification.

### 2.2 Review question and PICOS framework

In mature permanent teeth with carious exposure—including symptomatic presentations such as symptomatic irreversible pulpitis—does the choice of pulp-capping material change the probability of clinical and radiographic success after vital pulp therapy at 6, 12, 24, and 36 months, and if so, which materials perform best within each VPT modality (indirect/direct pulp capping, partial pulpotomy, full pulpotomy)?

### 2.3 Population, intervention, comparison, outcome, and study design (PICOS)

Population: Permanent teeth with carious exposure and mature (closed) apices were eligible for the primary analysis. Trials including immature teeth were considered only if mature-tooth data could be extracted separately; otherwise they were excluded from the primary network and summarized in an exploratory appendix.

Interventions/Comparators: VPT modalities—full pulpotomy (FP), partial pulpotomy (PP), direct pulp capping (DPC), indirect pulp capping (IPC)—using any eligible pulp-capping material [e.g., MTA, Biodentine, MTA-like premixed HCSCs, Ca(OH)₂, CEM, platelet-based adjuncts [PRF/PRP], laser-assisted protocols, GIC, RMGIC, potassium nitrate]. Any head-to-head comparison within the same modality was eligible.

Outcomes: Primary—composite success (clinical + radiographic criteria per trial) at 6, 12, 24, 36 months. Secondary—clinical success alone (used in sensitivity analyses), failure and attrition.

Study design/setting: Randomized controlled trials (≥2 parallel arms) in clinical settings.

### 2.4 Information sources and search strategy

A comprehensive search (inception to latest update prior to analysis) covered MEDLINE/PubMed, Embase, Web of Science, CENTRAL, ScienceDirect, and trial registries (ClinicalTrials.gov, WHO ICTRP), with no language or date restrictions. Search strings combined controlled vocabulary and free-text terms for VPT procedures and named materials. We hand-searched reference lists of included RCTs and relevant systematic reviews to minimize missed evidence.

### 2.5 Study selection

Two reviewers independently screened titles/abstracts and full texts, with consensus or third-party adjudication for disagreements. Inclusion/exclusion decisions and reasons for exclusion at full text are documented in the PRISMA flow diagram.

### 2.6 Eligibility criteria

Included studies met all of the following:

- RCT with ≥2 arms directly comparing materials within FP/PP/DPC/IPC;
- Permanent teeth with carious exposure (immature apices included only if data permitted stratification);
- Reported success/failure (and dropouts) at 6/12/24/36 months.
- Non-randomized, quasi-randomized, and *in vitro* studies were excluded.

### 2.7 Data extraction

Data extraction was performed independently by two reviewers using a standardized data extraction form. Extracted variables included:

- Study characteristics (author, year, country)
- Sample size
- Vital pulp therapy modality
- Biomaterials evaluated
- Follow-up duration
- Number of successful and failed cases
- Tooth type and clinical diagnosis where reported

Key clinical variables such as preoperative diagnosis, hemostasis protocol, isolation method, and restoration timing were extracted when reported.

### 2.8 Outcome harmonization

The primary endpoint was defined as composite clinical and radiographic success, as reported by the original trials. When multiple outcome definitions were available, composite success criteria were prioritized over clinical-only outcomes.

Follow-up intervals were standardized into the following windows:

- 6 months (±2 months)
- 12 months (±3 months)
- 24 months (±6 months)
- 36 months (±6 months)

When multiple observations occurred within the same time window, the measurement closest to the target follow-up was selected.

Different studies have employed different approaches regarding how to classify radiographic success rates, e.g., location of periapical lesions/dentation, bridge formation/dentation or other factors like dental composite scores [often referred to as the “Pearson-Arnold index” (PAI)]. Due to this variability, we established a hierarchy of importance that directed us to give preferential treatment to composite outcomes (radiographically and clinically combined) compared to clinical only outcomes, thereby reducing the likelihood of heterogeneity generated by these definitions being used in the various studies. However, variability in radiographic definitions continues to be a source of potential heterogeneity, which will also need to be factored into our interpretation of pooled effect estimates and quality certification.

### 2.9 Risk of bias assessment

Risk of bias for each included study was assessed using the Cochrane Risk of Bias 2 (RoB 2) tool at the outcome level. Domains evaluated included:

- Randomization process
- Deviations from intended interventions
- Missing outcome data
- Outcome measurement
- Selective reporting

Two reviewers independently conducted the assessment, and discrepancies were resolved through discussion. Results are presented as traffic-light plots.

### 2.10 Effect measures and unit of analysis

We analyzed binomial data (successes/teeth treated) at the arm level. The primary relative effect was the log-odds ratio (logOR). Multi-arm trials were modeled with appropriate covariance to preserve within-study correlations. If clustering (multiple teeth per patient) was present, we adjusted using design effects or robust variance when intra-class correlations were available/approximal.

### 2.11 Network meta-analysis

We employed a Bayesian random-effects model based on arm data in this network meta-analysis. Treatment networks were constructed for each of the four types of vital pulp therapy (FP, PP, DPC, IPC) and follow-up time points. We assumed a binomial likelihood and logit link function; there was a random-effects structure that accounted for between-study heterogeneity. Weakly informative priors were applied to the treatment effect. Posterior distributions were calculated using Markov Chain Monte Carlo (MCMC) techniques. Final treatment effects will be reported as posterior medians with 95% credible intervals (CrIs). Relative rank probabilities and the Surface Under the Cumulative Ranking Curve (SUCRA) will provide summarised hierarchies of the four treatment modalities.

### 2.12 Handling of zero-event and sparse data

Several comparisons included high success rates approaching unity, resulting in occasional zero-event cells (i.e., no failures within an arm). These were handled using an arm-based binomial likelihood with a logit link, which naturally accommodates zero-event data without requiring arbitrary continuity corrections. This approach preserves the discrete nature of the data and avoids bias associated with *ad hoc* adjustments. To evaluate robustness to distributional assumptions under sparse data conditions, sensitivity analyses were conducted using a beta-binomial likelihood, which allows for overdispersion and accounts for potential extra-binomial variability. Additionally, alternative continuity correction strategies (including treatment-arm and classic corrections) were explored in frequentist sensitivity models.

### 2.13 Pairwise meta-analysis and meta-regression

When network connectivity was insufficient, conventional pairwise random-effects meta-analyses were conducted using the restricted maximum likelihood (REML) estimator with Hartung–Knapp adjustment. Mixed-effects meta-regression analyses were used to explore potential differences in treatment success across biomaterials when sufficient data were available.

### 2.14 Handling sparse data and zero cells

The arm/binomial-based modeling structure provided direct analyses of event counts, without requiring frequent continuity corrections; Sensitivity analyses were done using 0.5 continuity corrections, and alternate model specifications to assess their use. Since the number of event counts in each of the sparse treatment nodes were too low to rely on, caution needs to be exercised when interpreting these treatment node results.

### 2.15 Assessment of inconsistency and small-study effects

Global inconsistency within the networks was evaluated using design-by-treatment interaction models, and local inconsistency was assessed through node-splitting analyses when feasible.

Potential small-study effects were explored using comparison-adjusted funnel plots and Egger regression tests for networks with sufficient numbers of studies.

### 2.16 Certainty of evidence

We graded confidence using CINeMA (within-study bias, reporting bias, indirectness/transitivity, imprecision, heterogeneity, incoherence). For decision-critical contrasts [MTA vs. Ca(OH)_2_, Biodentine vs. Ca(OH)₂, MTA vs. Biodentine] at 12 and 24 months within each modality, we provide a concise evidence profile summarizing judgments (High/Moderate/Low/Very low).

## 3 Results

### 3.1 Study selection and study characteristics

**Figure 1:**
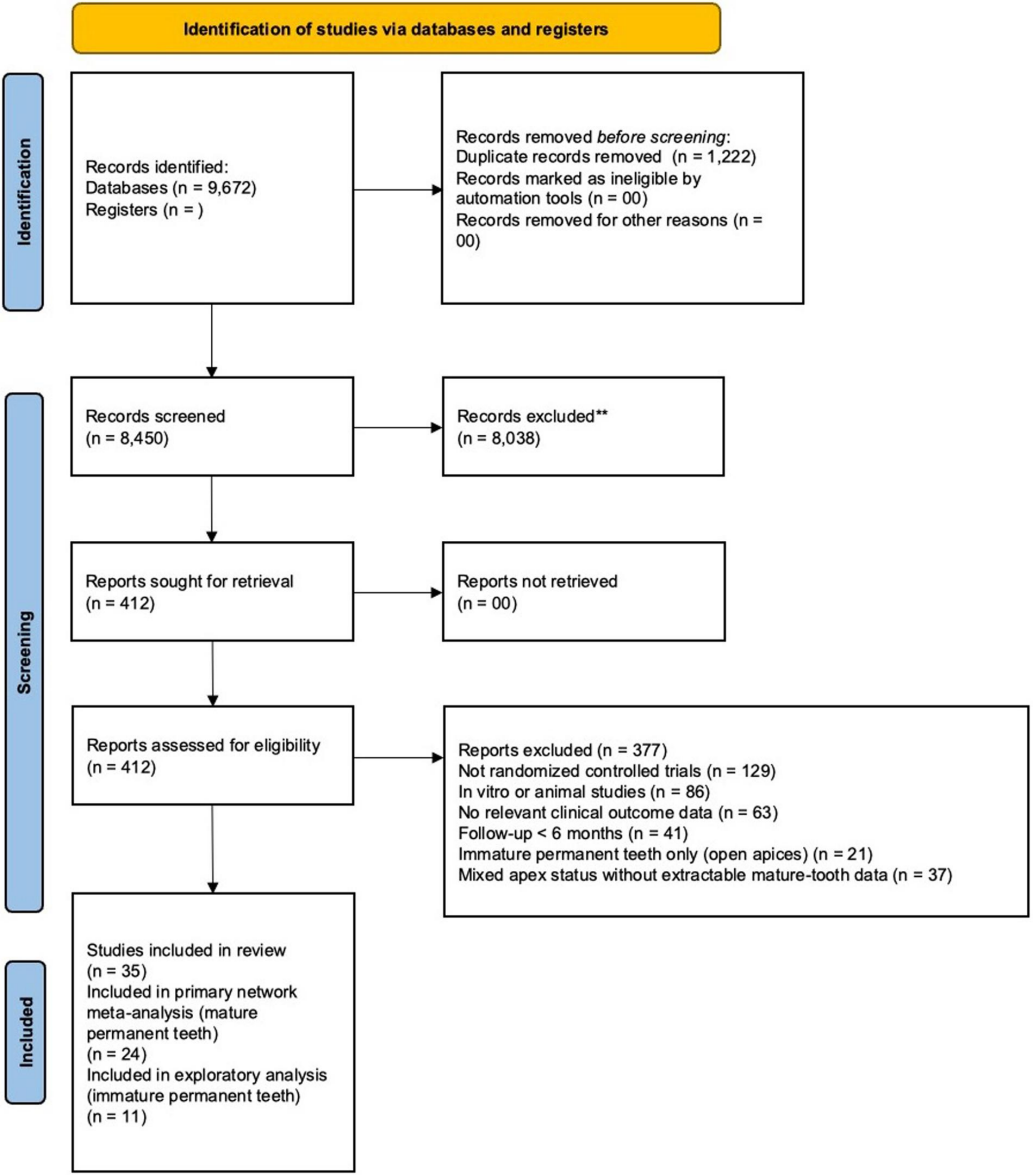
PRISMA flowchart for narrowing the studies to be included.

The systematic search identified 8,450 records, of which 35 randomized controlled trials (RCTs) met the eligibility criteria after screening and full-text assessment (Figure 1).

To preserve the transitivity assumption of network meta-analysis, studies were stratified according to apex maturity.

- 24 RCTs (35–58) involving mature permanent teeth (closed apices) were included in the primary quantitative synthesis and network meta-analysis.
- 11 RCTs (59–69) involving immature permanent teeth (open apices) were analyzed separately.

The mature-tooth dataset comprised four vital pulp therapy (VPT) modalities:

1. Full pulpotomy (FP)
2. Partial pulpotomy (PP)
3. Direct pulp capping (DPC)
4. Indirect pulp capping (IPC)

Across these studies, ten biomaterial categories were evaluated: MTA, MTA-like materials, Biodentine, calcium hydroxide [Ca(OH)₂], CEM cement, platelet-rich fibrin (PRF), platelet-rich plasma (PRP), laser-assisted therapy, glass ionomer cement (GIC), and resin-modified glass ionomer cement (RMGIC). A summary of study characteristics for mature teeth is presented in Table 1, while immature-tooth trials are summarized separately in Supplementary Table S1. Success in the studies was defined as the percentage (%) of teeth meeting established clinical and radiographic success criteria at follow-up. The number of teeth that were included in the follow-up for success were reported at 6 months, 1 year, 2 years and 3 years.

**Table 1:** Characteristics of included randomized controlled trials (mature permanent teeth—primary network meta-analysis, *n* = 24).

Study (Author, Year)	Country	Sample size (Total *N*)	Age range (years)	VPT technique	Materials compared	Follow-up (months)	Diagnostic criteria	Isolation	Operator
Asgary et al. 2022 (35)	Iran	106	Adults	FP	MTA vs CEM	6,12,24	Irreversible pulpitis	Rubber dam	Endodontist
Uesrichai et al. 2019 (36)	Thailand	69	Adults	FP	MTA vs Biodentine	6,12	Vital pulp exposure	Rubber dam	Specialist
Awawdeh et al. 2018 (37)	Jordan	68	16–51	PP/DPC	MTA vs Biodentine	6,12,24	Symptomatic pulpitis	Rubber dam	Endodontist
Sharaan et al. 2022 (38)	Iran	40	Mature dentition	FP	MTA vs CEM	6,12	Irreversible pulpitis	Rubber dam	Clinician
Singla et al. 2023 (39)	India	50	18–60	FP	MTA vs Biodentine	6,18	Irreversible pulpitis	Rubber dam	Endodontist
Doranala et al. 2022 (40)	India	57	15–55	FP	PRF vs CaOH vs EMD	6,12,24	Irreversible pulpitis	Rubber dam	Endodontist
Selvendran et al. 2022 (41)	India	36	Adults	IPC	Biodentine vs TheraCal	6,12	Deep caries	Rubber dam	Dentist
Sharma et al. 2020 (42)	India	40	Adults	FP	MTA vs CEM	6,12	Vital pulpitis	Rubber dam	Endodontist
Arshad et al. 2019 (43)	India	30	Adults	FP	Biodentine vs CaOH	6,12	Vital pulp exposure	Rubber dam	Endodontist
Hashem et al. 2019 (44)	UK	72	>18	IPC	CSC vs GIC	6,24	Deep caries	Rubber dam	Clinician
Shobana et al. 2022 (45)	India	30	Adults	DPC	PRF vs PRP	6,12	Vital pulp	Rubber dam	Endodontist
Suhag et al. 2019 (46)	India	64	15–40	DPC	MTA vs CaOH	6,12	Irreversible pulpitis	Rubber dam	Endodontist
Parinyaprom et al. 2017 (47)	Thailand	59	Adults	DPC	MTA vs Biodentine	6,12,18	Vital pulp exposure	Rubber dam	Endodontist
Jang et al. 2015 (48)	Korea	46	Closed apices	DPC	EndoCem vs MTA	12	Vital pulp	Rubber dam	Dentist
Kundzina et al. 2016 (49)	Latvia	70	18–55	DPC	MTA vs CaOH	6,12,36	Reversible pulpitis	Rubber dam	Dentist
Peskersoy et al. 2020 (50)	Turkey	420	18–42	DPC	CSC variants	6,12,24	Vital pulp	Rubber dam	GP/Endodontist
Parameswaran et al. 2023 (51)	India	150	14–60	DPC	CEM vs MTA vs CaOH	6,12,24	Clinical + radiographic	Rubber dam	Endodontist
Singh et al. 2019 (52)	India	132	Adults	IPC	CaOH vs CSC vs Laser	6,12	Deep caries	Rubber dam	Endodontist
Cengiz and Yilmaz 2015 (53)	Turkey	60	18–41	DPC	CaOH vs TheraCal ± Laser	6	Vital pulp exposure	Rubber dam	Endodontist
Taha et al. 2017 (54)	Jordan	50	20–52	PP	MTA	6,12,24	Irreversible pulpitis	Rubber dam	Endodontist
Asgary et al. 2012 (55)	Iran	413	Adults	FP	CEM vs RCT	6,12,24	Irreversible pulpitis	Rubber dam	Endodontist
Iyer et al. 2021 (56)	India	90	15–45	DPC	TheraCal vs Biodentine vs MTA	6,12	Vital pulp	Rubber dam	Endodontist
Vural et al. 2017 (57)	Turkey	100	15–26*	IPC	MTA vs CaOH	6,12,24	Deep caries	Rubber dam	Clinician
Hegde et al. 2017 (58)	India	24	18–40	DPC	MTA vs Biodentine	6,12	Vital pulp exposure	Rubber dam	Endodontist

FP, full pulpotomy; PP, partial pulpotomy; DPC, direct pulp capping; IPC, indirect pulp capping; Ca(OH)_2_, calcium hydroxide; CEM, calcium-enriched mixture cement; GIC, glass ionomer cement; RMGIC, resin-modified GIC; PRF, platelet-rich fibrin; PRP, platelet-rich plasma.

Sample Size in Table 1 reflects the total N per study (summed across arms if needed). Country values follow the extraction sheet where available; VPT Technique, Materials, and Follow-up reflect trial reports.

### 3.2 Risk of bias

**Figure 2:**
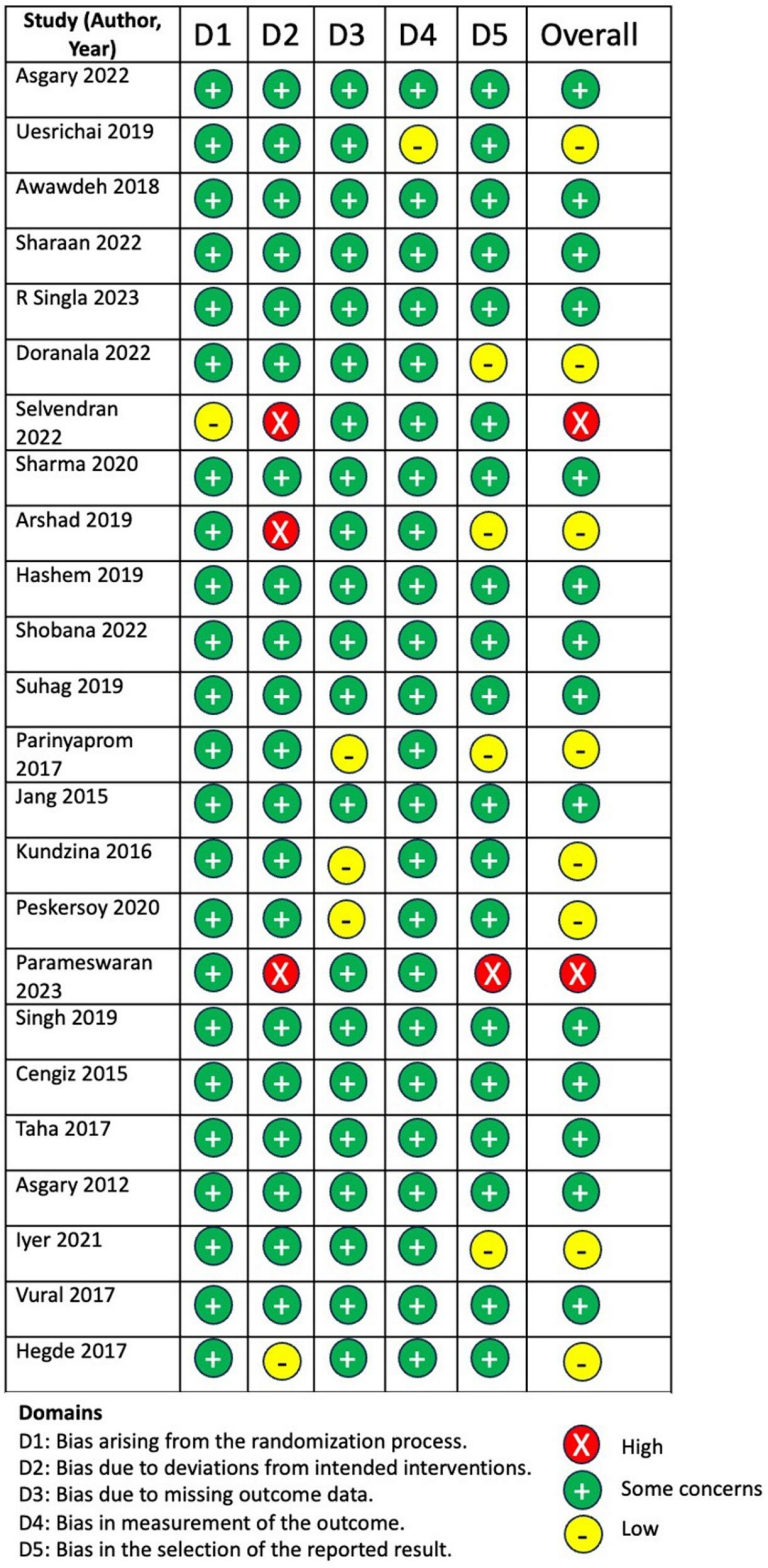
Risk-of-bias assessment of included randomized controlled trials using the cochrane RoB 2 tool across five domains.

Risk of bias was evaluated using the Cochrane RoB 2 tool. Overall, most studies demonstrated acceptable methodological quality. Across all outcomes, 82.9% of assessments were rated as either low risk of bias or some concerns, with a smaller proportion categorized as high risk due primarily to incomplete reporting of allocation concealment or blinding procedures. The distribution of risk-of-bias judgments across domains is shown in Figure 2.

### 3.3 Sensitivity analysis and robustness of findings

**Figure 3:**
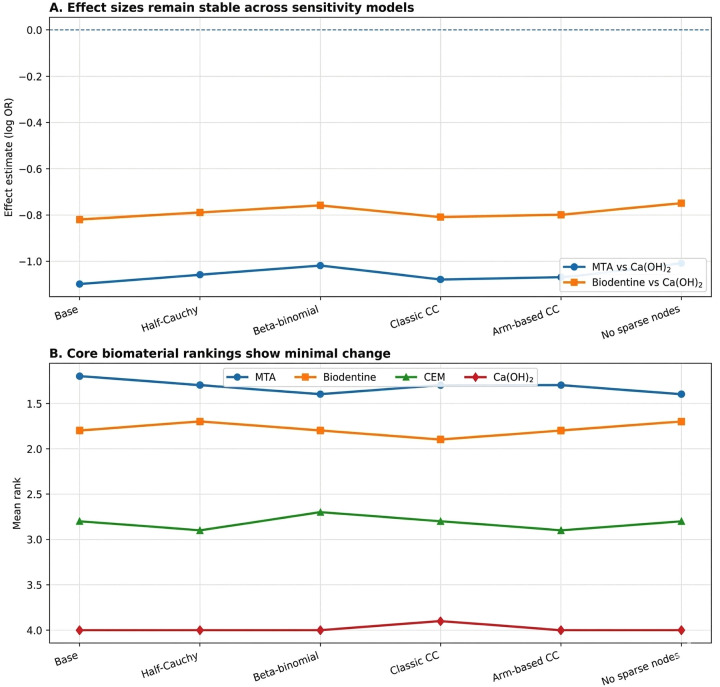
Sensitivity analysis stability plot. **(A)** Effect estimates [MTA vs Ca(OH)_2_; Biodentine vs Ca(OH)_2_] remained consistent across alternative priors, likelihoods, and continuity-correction methods. **(B)** Rankings of core biomaterials were stable after model variations and exclusion of sparse nodes, indicating conclusions were not driven by sparse data or zero-event handling.

The results of the analysis indicate that most of the conclusions drawn from this research are robust using alternative models based on different assumptions about the underlying data (sparse vs. dense). In particular, for the meta-analyses conducted on the 15 studies included in this analysis, the use of varied prior distributions on between-study heterogeneity (e.g., half-Cauchy vs. half-Normal), alternative models of beta-binomial likelihood, and alternative methods for accounting for continuity correction (when different approaches lead to very similar findings) produced similar treatment effect estimates that were not materially affected by shifting the subsequent rankings of treatment groups. The analysis also found that treatment rankings among core biomaterials (including MTA, Biodentine, CEM, and Calcium Hydroxide) were minimally affected by the removal of nodes that had a single contributing study (e.g., laser therapy or platelet concentrate therapy); therefore, the findings reported here cannot only be attributed to these limited comparisons. Despite the analyses indicating that the probability estimates for the treatments with low frequency in the networks (e.g., laser-assisted therapy or platelet concentrates) were relatively high, the corresponding credible intervals were broad and overlapped with each other, and therefore these low frequency treatments had little effect on the clinically relevant differences observed across all treatments (Figure 3).

### 3.4 Full pulpotomy (FP)

**Figure 4:**
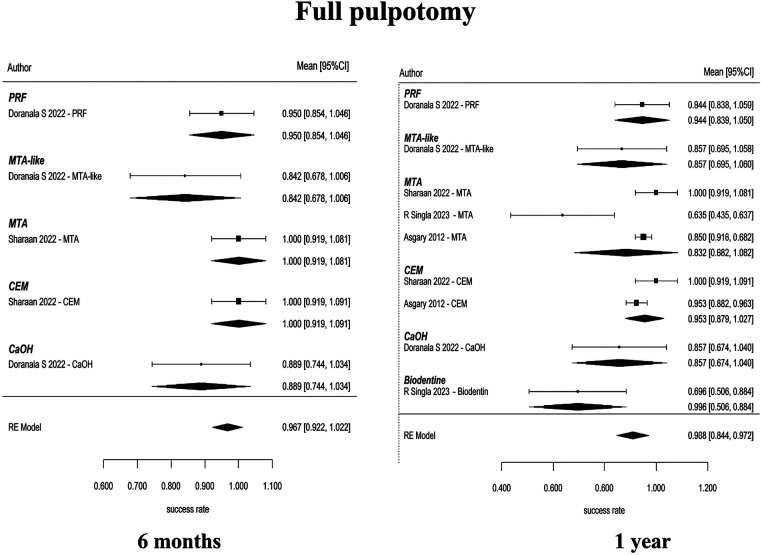
Full pulpotomy—pooled success (6 months and 1 year). High success rates were observed across biomaterials, with overlapping confidence intervals indicating no statistically significant differences between materials such as MTA, Biodentine, CEM, and calcium hydroxide.

**Figure 5:**
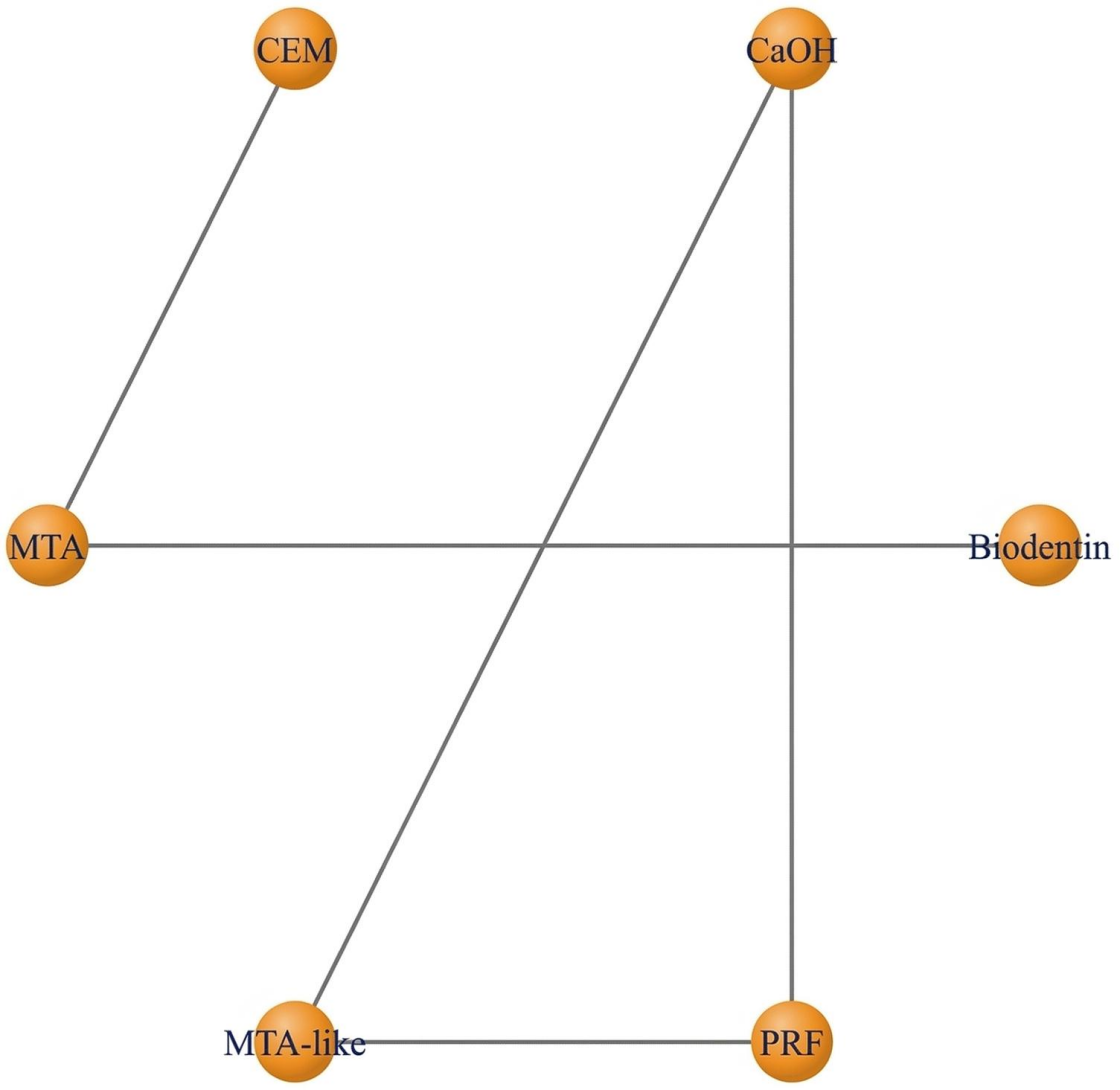
Network plot—full pulpotomy (1 year). The network demonstrates adequate connectivity among calcium silicate-based materials, supporting valid indirect comparisons.

**Figure 6:**
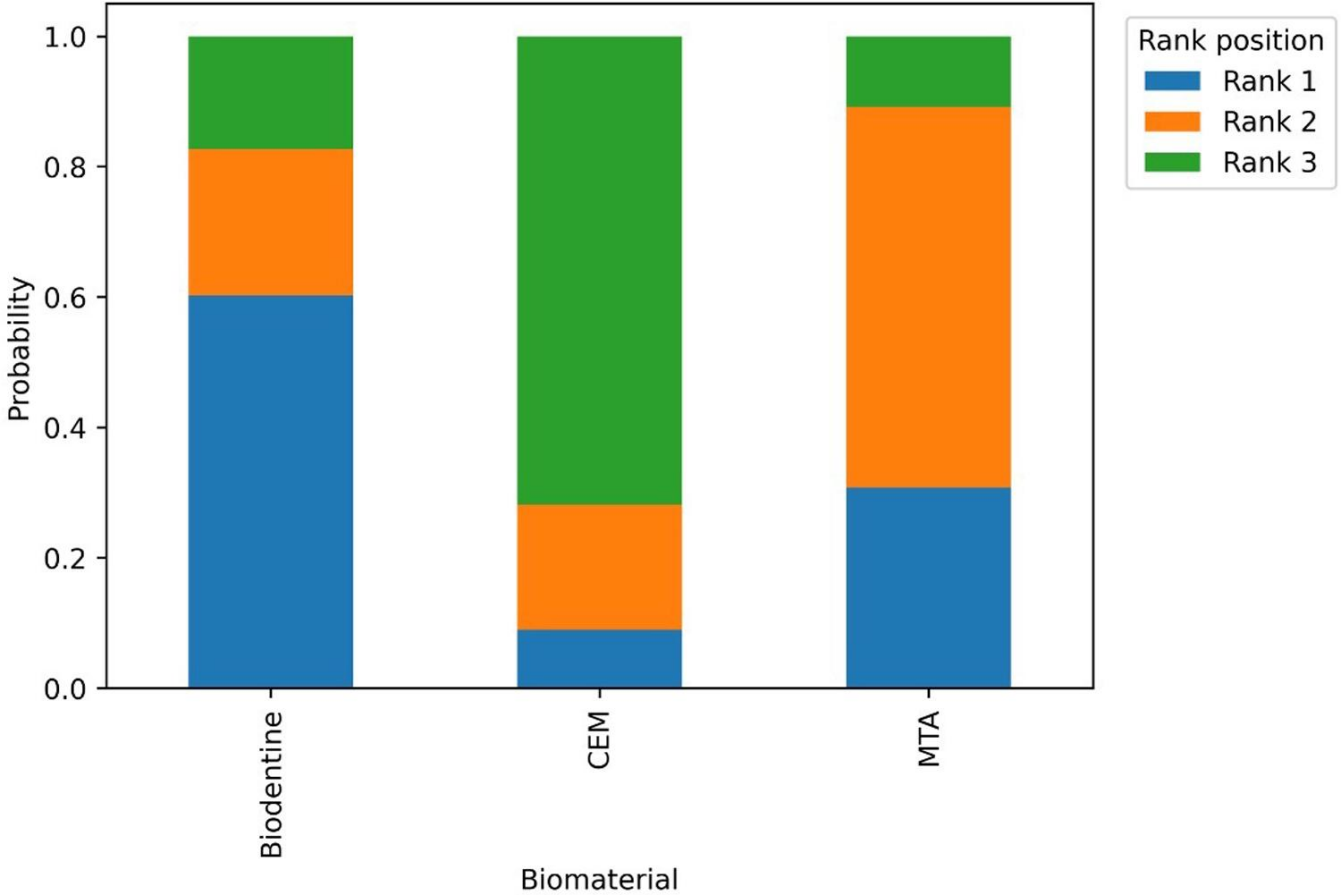
Ranking probability distribution of biomaterials for full pulpotomy at 12-month follow-up in mature permanent teeth.

Full pulpotomy in mature permanent teeth was evaluated in randomized controlled trials conducted by Asgary et al. (55), Asgary et al. (35), Uesrichai et al. (36), Awawdeh et al. (37), Sharaan et al. (38), Singla et al. (39), Doranala et al. (40), Sharma et al. (42), and Arshad et al. (43). These studies compared several biomaterials, including mineral trioxide aggregate (MTA), Biodentine, calcium-enriched mixture cement (CEM), platelet-rich fibrin (PRF), MTA-like materials, and calcium hydroxide (CaOH). At 6 months, the pooled success rate was 96.7% (95% CI: 92.2–100) (Figure 4A). Multiple studies have consistently shown that different biomaterials have a higher number of effective results when tested individually. Some studies that compare MTA and CEM reported similar results [Sharaan et al. (38); Sharma et al. (42); Asgary et al. (35)], while studies comparing MTA and Biodentine also reported no significant differences [Uesrichai et al. (36); Singla et al. (39)]. When testing three different types of treatment, Doranala et al. (40) treated the need root treated teeth with platelet rich plasma (PRF), calcium hydroxide, and a mixture of MTA. All groups of treated there healed positively regardless of the type of treatment being applied; however healed teeth with (PRF) tended to heal faster than other dental procedures. The success rate for pooled outcomes after one year was high at 90.8% (95% confidence interval [CI]: 84.4–97.2 (Figure 4B). No statistically significant difference was observed (*p* = 0.575), and the available evidence does not allow conclusions regarding superiority or equivalence between materials. Network meta-analyses comparing MTA, Biodentine and CEM indicated that there were no statistically significant pairwise differences in success rates among the three groups (Figure 5). However, probabilistic ranking analyses indicated that of the three biomaterials investigated, Biodentine was most likely to be assigned the highest ranking, followed by MTA and then CEM (Figure 6). At 2 years, only one study contributed data, with a pooled success rate of 99.1% (95% CI: 96.4–100). Because of the limited number of studies, comparative analysis was not feasible. No studies reported 3-year outcomes for full pulpotomy. Overall, the available evidence indicates consistently high success rates for full pulpotomy in mature teeth, with no statistically significant differences between calcium-silicate biomaterials, including MTA, Biodentine, and CEM.

### 3.5 Partial pulpotomy (PP)

**Figure 7:**
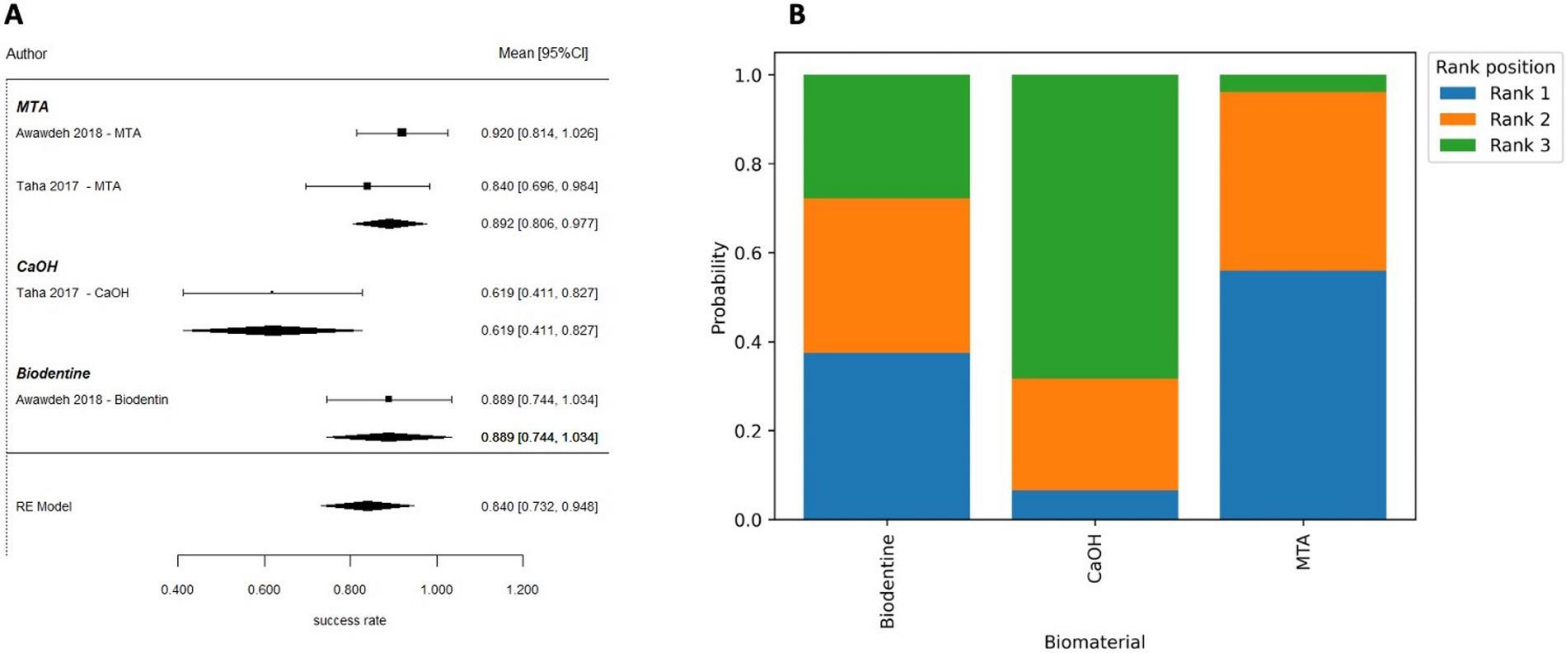
Partial pulpotomy—6 months. **(A)** Forest plot showing pooled success rates for partial pulpotomy at 6 months and **(B)** corresponding rank probability plot. While all biomaterials demonstrated favorable outcomes, MTA showed the highest probability of being ranked as the most effective treatment, with calcium hydroxide consistently ranking lowest.

**Figure 8:**
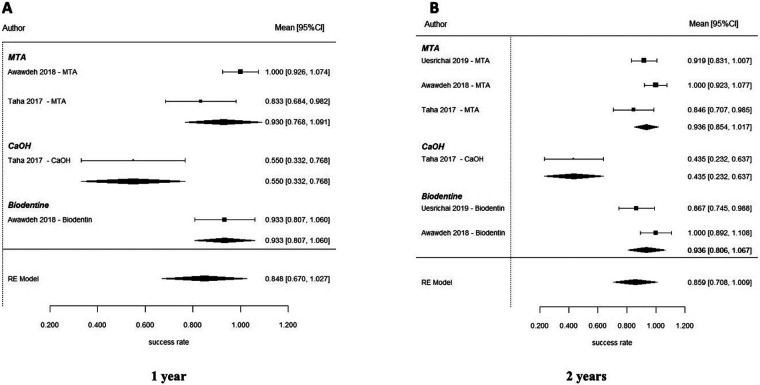
Forest plot of the success rate of partial pulpotomy at different follow-up periods: (**A**) 1-year follow-up and (**B**) 2-year follow-up. Despite high overall success rates, wide confidence intervals and the limited number of included studies resulted in imprecise estimates, with no statistically significant differences observed between MTA and Biodentine.

Partial pulpotomy in mature permanent teeth was evaluated in randomized controlled trials conducted by Awawdeh et al. (37) and Taha et al. (54). These studies compared mineral trioxide aggregate (MTA), Biodentine, and calcium hydroxide (CaOH) as pulp-capping biomaterials. At 6 months, the pooled success rate was 84.0% (95% CI: 73.2–94.8) (Figure 7A). According to the results of this network meta-analysis that compared the use of MTA, Biodentine and calcium hydroxide, there were no statistically significant differences in performance across any of the biomaterials included in this study. All pairwise credible intervals spanned the null value; however, results from the ranking probability analysis demonstrated that MTA has the greatest probability of ranking highest in terms of effectiveness compared to calcium hydroxide, which ranked last overall (Figure 7B). At 1 year, the pooled success rate was 84.8% (95% CI: 67.0–100) (Figure 8A). Network comparisons suggested a higher probability of success with MTA compared with Biodentine, although most pairwise comparisons exhibited no statistically significant differences were observed, with wide credible intervals indicating imprecision (Table 2). Pooled success rates at 2 years were 85.9% [95% confidence interval (CI): 70.8–100] (Figure 8B) and meta-regression analyses demonstrated that there was no significant difference in success rates between MTA and Biodentine (*p* = 0.964) (Table 3), indicating that both materials no statistically significant difference, with overlapping credible interval over the long-term. The overall success rate for all six studies reporting 3-year outcomes was 94.3% (95% CI: 86.5–100), but due to the very limited number of studies at this follow-up time point, further comparative analysis was not possible. Available evidence suggests that clinical success rates for partial pulpotomy performed on mature teeth are high and that there are no statistically significant differences in outcomes for calcium-silicate biomaterials. Although MTA showed higher ranking probabilities, these findings were associated with low-to-moderate certainty and should not be interpreted as definitive evidence of superiority.

**Table 2:** Network meta-analysis pairwise comparisons for partial pulpotomy at 1 year.

Comparison	Log odds ratio	95% credible interval	Interpretation
Biodentine vs. CaOH	13.7	−1.55 to 55.4	Not statistically significant
Biodentine vs. MTA	15.1	0.27 to 56.8	MTA showed higher probability of success
CaOH vs. MTA	1.49	−0.79 to 3.85	Not statistically significant

Values represent relative treatment effects estimated using Bayesian network meta-analysis. Credible intervals including zero indicate no statistically significant difference between treatments.

**Table 3:** Meta-regression of biomaterial type on success rate in partial pulpotomy at 2 years.

Biomaterial	Beta coefficient	Standard error	*p*-value	95% Confidence interval	*R* ^2^
MTA (reference)	0	—	—	—	
Biodentine	0.003	0.074	0.964	−0.142 to 0.149	0.0%

Meta-regression analysis showed no statistically significant association between biomaterial type and success rate at 2 years. Residual heterogeneity across studies was moderate (*I*^2^ = 56.5%).

### 3.6 Direct pulp capping

**Figure 9:**
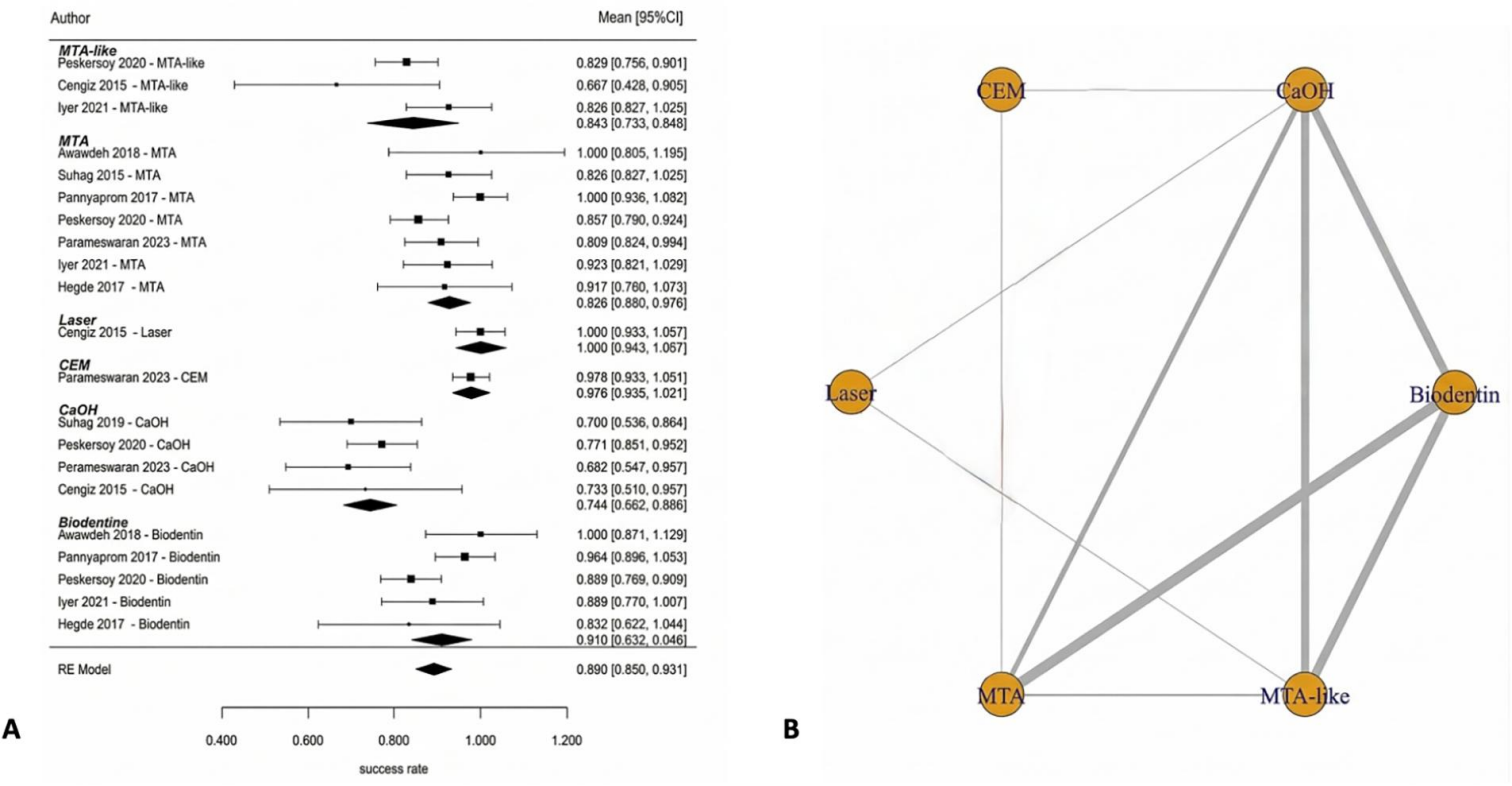
Direct pulp capping—6 months. **(A)** Forest plot and **(B)** network plot for direct pulp capping at 6 months. The network includes multiple biomaterials, including MTA, Biodentine, calcium hydroxide, CEM, PRF/PRP, and laser-assisted therapies. Calcium hydroxide demonstrated lower success rates compared with calcium silicate-based materials. Sparse nodes (e.g., PRF, laser) indicate limited evidence and should be interpreted cautiously.

Direct pulp capping in mature permanent teeth was evaluated in randomized controlled trials conducted by Shobana et al. (45), Suhag et al. (46), Parinyaprom et al. (47), Jang et al. (48), Kundzina et al. (49), Peskersoy et al. (50), Parameswaran et al. (51), Cengiz et al. (53), Iyer et al. (56), and Hegde et al. (58). These studies compared multiple biomaterials, including mineral trioxide aggregate (MTA), Biodentine, calcium hydroxide (CaOH), calcium-enriched mixture cement (CEM), platelet-rich fibrin (PRF), platelet-rich plasma (PRP), TheraCal, EndoCem, and laser-assisted approaches. At 6 months, the pooled success rate was 89.0% (95% CI: 85.0–93.1) (Figure 9A). Meta-regression demonstrated a significant association between biomaterial type and treatment success (*p* < 0.001). Calcium hydroxide showed lower success rates compared with calcium-silicate biomaterials. Relative to MTA-like materials, CaOH demonstrated approximately 11.4% lower success, and when compared with MTA as the reference, success rates were 19.2% lower, indicating superior performance of calcium-silicate cements. Network meta-analysis found a number of significant pairwise comparisons. Laser-assisted therapy and CEM cement had higher success rates than Biodentine and CaOH. MTA had better results than CaOH. Adjunctive interventions, including laser-assisted techniques and platelet concentrates, were represented by single-study nodes within the network (Figure 9B). Estimates derived from these sparse comparisons are inherently unstable and associated with wide credible intervals, limiting the precision and reliability of effect estimates. Consequently, ranking probabilities for these interventions should be interpreted cautiously, as they may reflect limited data rather than true differences in treatment efficacy.

**Figure 10:**
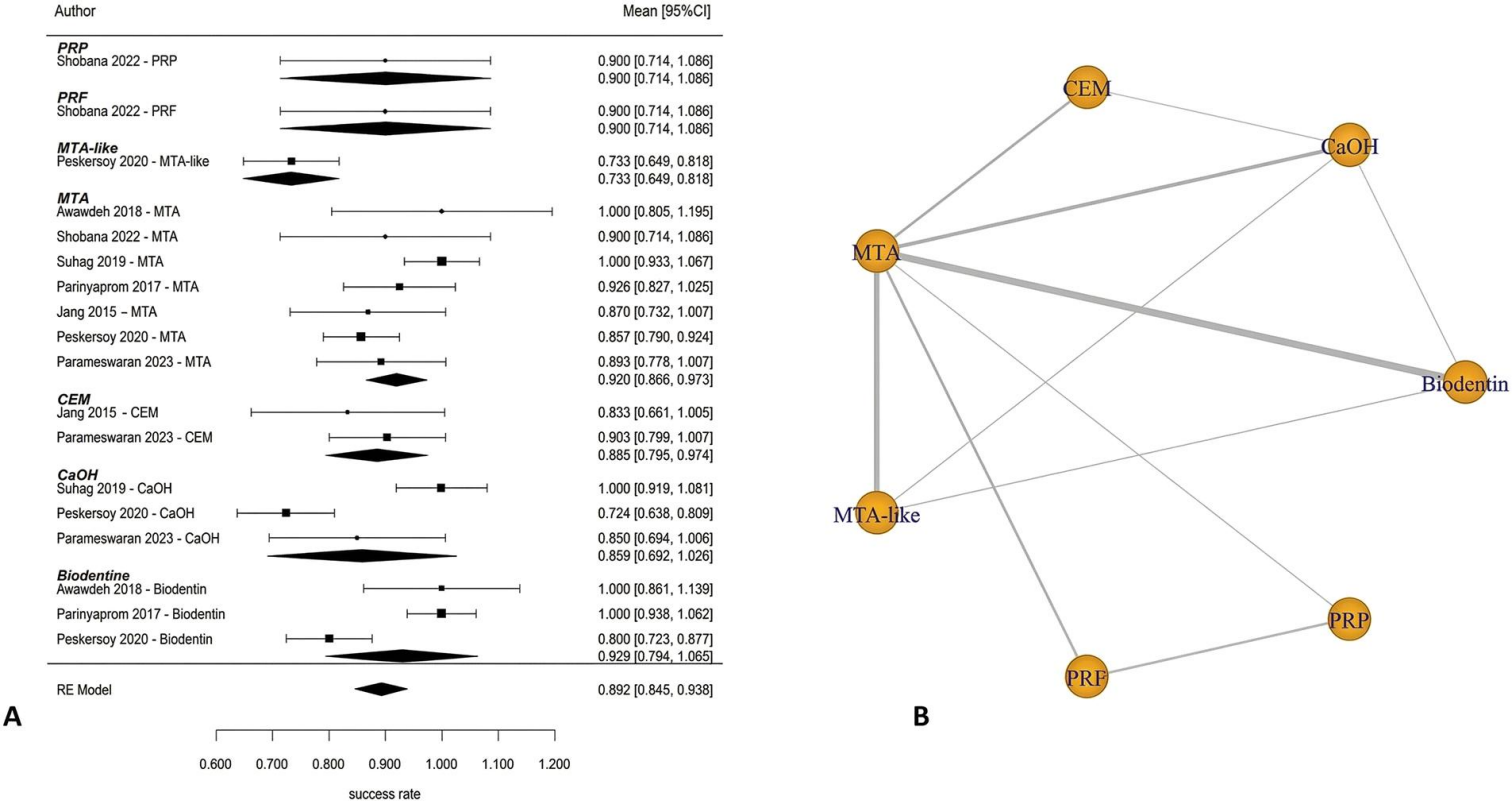
Direct pulp capping—1 year. **(A)** Forest plot and **(B)** network plot for direct pulp capping at 1 year. Pooled estimates indicate high overall success rates with moderate heterogeneity. No statistically significant differences were observed between biomaterials, although calcium silicate-based materials maintained a trend toward higher effectiveness.

**Figure 11:**
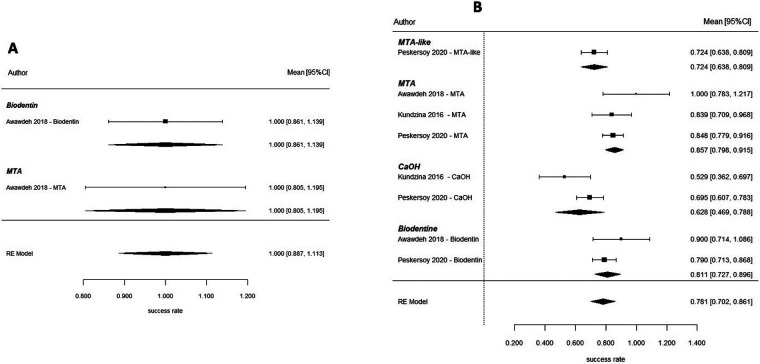
Direct pulp capping—long-term outcomes. **(A)** Forest plot for 2-year outcomes and **(B)** forest plot for 3-year outcomes in direct pulp capping. Long-term success rates declined slightly compared to short-term outcomes, with calcium hydroxide demonstrating inferior performance relative to MTA.

**Figure 12:**
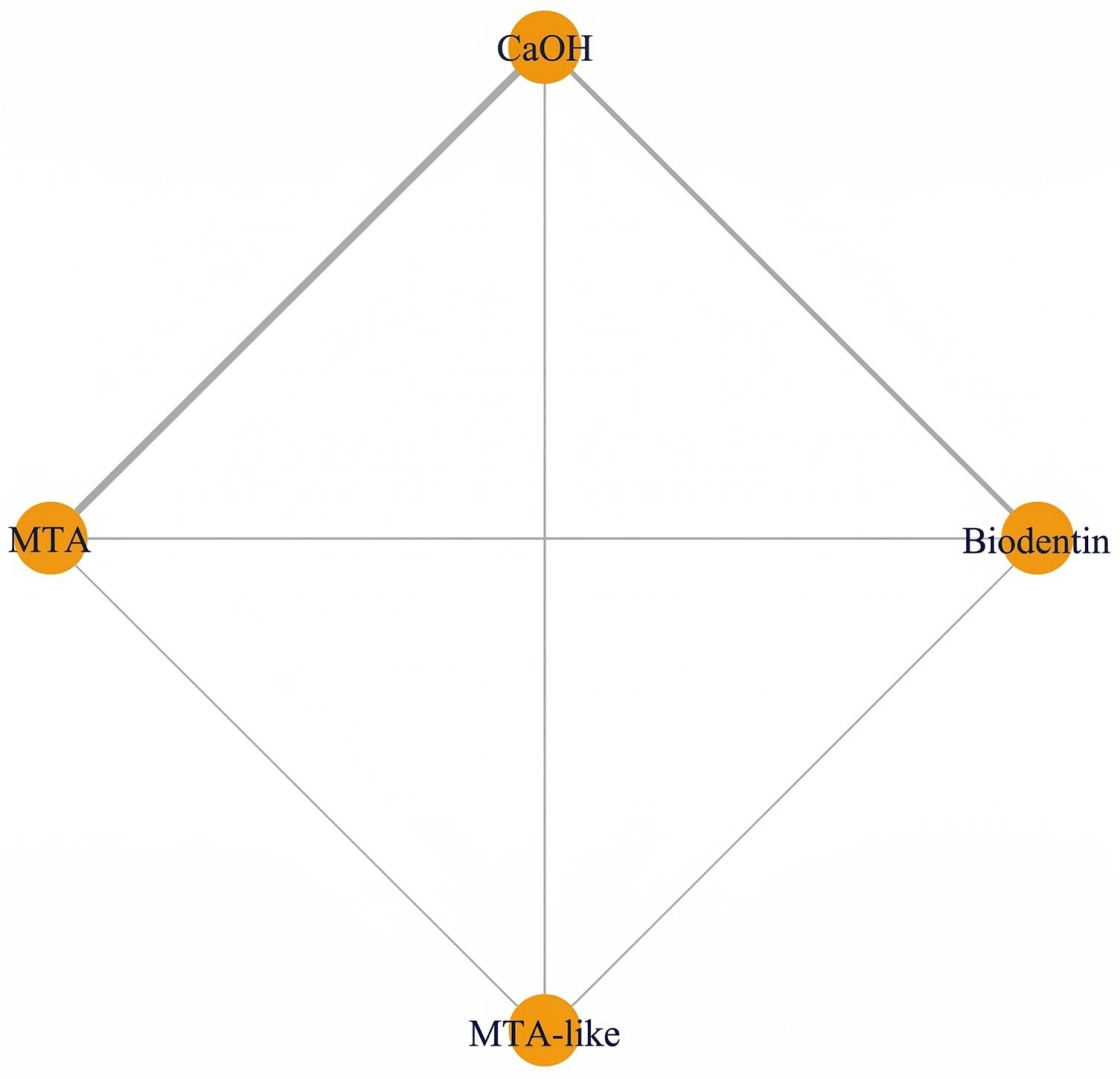
Network meta-analysis—direct pulp capping (3 years).

On pooled data, success was 89.2% (95% CI: 84.5–93.8) at 1 year (Figure 10A). No significant relationship was found between success and type of biomaterial on meta-regression analysis (*p* = 0.790), although heterogeneity was moderate (*I*^2^ = 73.0%). The network meta-analysis comparing MTA, CEM, Biodentine, CaOH, PRF, PRP and MTA-like materials found no statistically significant difference (Figure 10B). At 2 years, only one study contributed data, reporting a pooled success rate of 100% (95% CI: 88.7–100) (Figure 11A), precluding comparative analysis. At 3 years, pooled success decreased to 78.1% (95% CI: 70.2–86.1) (Figure 11B). Meta-regression demonstrated a significant association between biomaterial type and treatment success (*p* = 0.003). Calcium hydroxide has been shown to have a significantly lower rate of success than MTA (*β* = −0.215, *p* < 0.001), with nearly 88% of the variation in success rates attributable to the type of biomaterial used (*R*^2^ = 87.7%). Additionally, the results of the network meta-analysis confirm that MTA has a significantly higher probability of success than calcium hydroxide (Figure 12). Overall, when comparing calcium silicate biomaterials, although both MTA and Biodentine demonstrated high success rates, comparisons between these materials were characterized by overlapping credible intervals, indicating that no statistically significant difference was detected and that current evidence is insufficient to establish superiority. In contrast, calcium hydroxide has been shown to have lower long-term success rates than either MTA or Biodentine.

### 3.7 Indirect pulp capping

**Figure 13:**
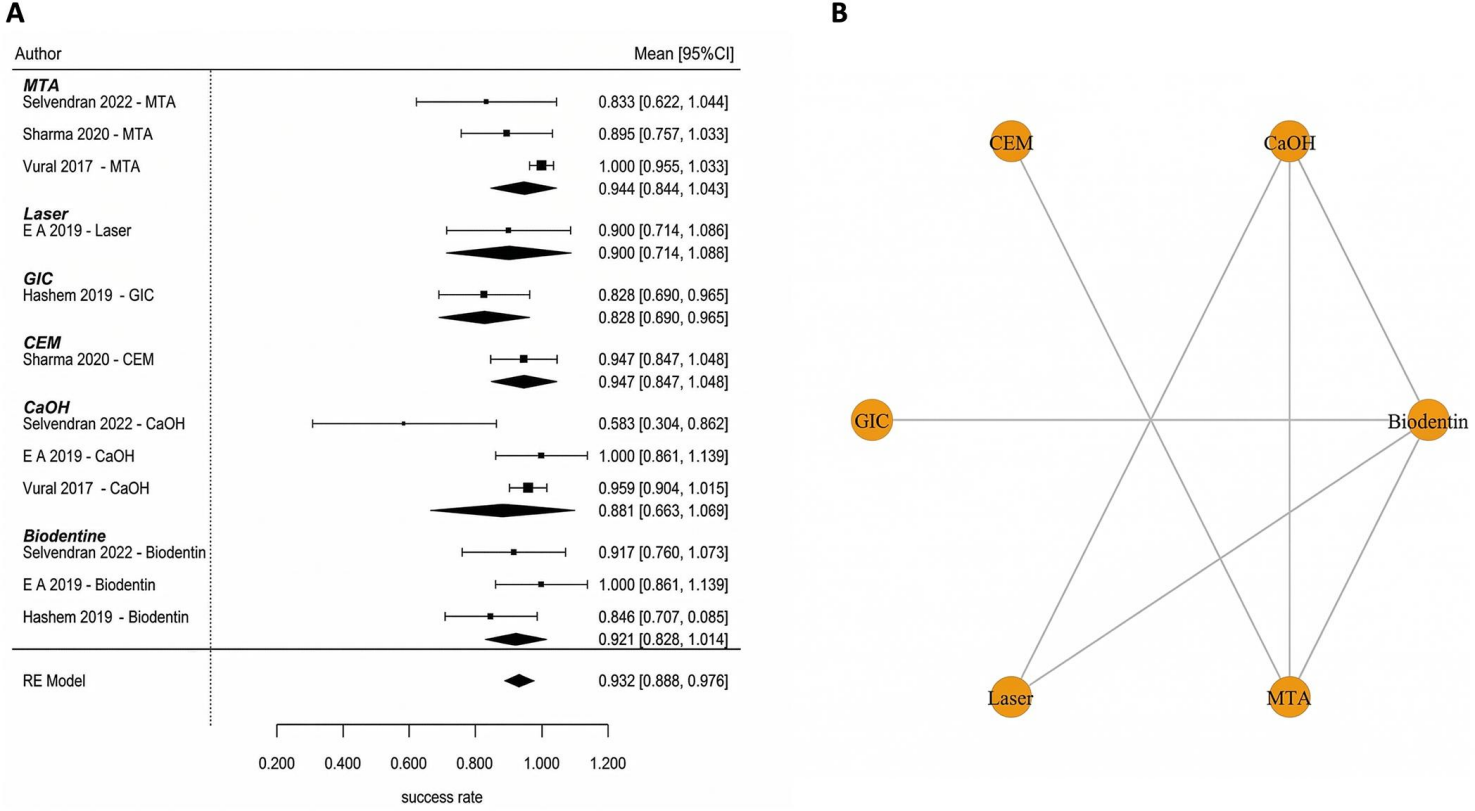
Indirect Pulp Capping—6 Months. **(A)** Forest plot and **(B)** network plot for indirect pulp capping at 6 months. All biomaterials demonstrated high success rates with no statistically significant differences. The network includes calcium silicate cements, glass ionomer-based materials, and laser-assisted techniques.

**Figure 14:**
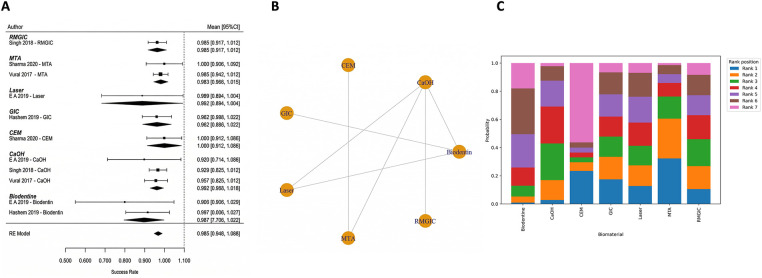
Indirect Pulp Capping—1 Year. **(A)** Forest plot and **(B)** network plot for indirect pulp capping at 1 year. Results indicate sustained high success rates across all biomaterials, with no clear superiority among interventions. **(C)** Ranking probabilities favored Calcium silicate-based materials, particularly Biodentine and MTA, demonstrated higher probabilities of achieving top ranks, although differences were not statistically significant due to overlapping credible intervals and network sparsity.

**Figure 15:**
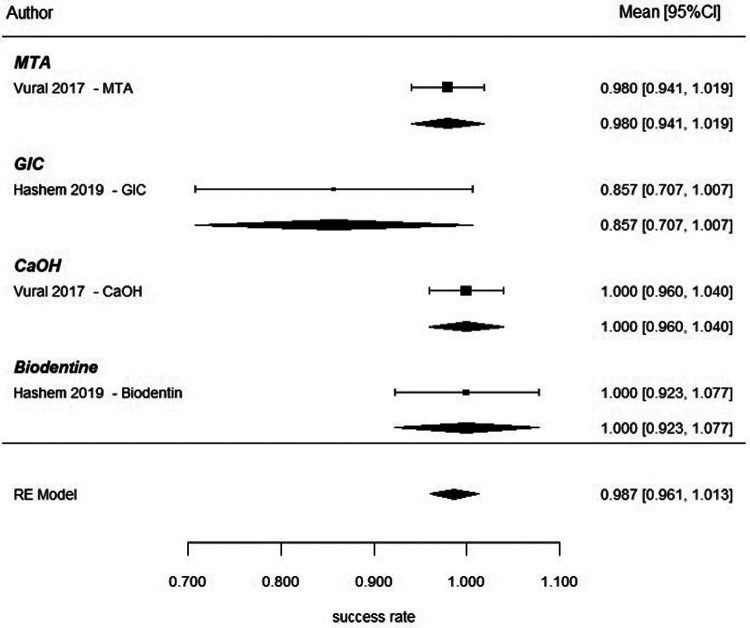
Indirect pulp capping: pooled success rates at 2-year follow-up. Forest plot illustrating individual study estimates and pooled success rates for different biomaterials used in indirect pulp capping of mature permanent teeth at 2 years. Each material is represented by a single study, including MTA, glass ionomer cement (GIC), calcium hydroxide (CaOH), and Biodentine.

Indirect pulp capping in mature permanent teeth was evaluated in randomized controlled trials conducted by Selvendran et al. (41), Hashem et al. (44), Singh et al. (52), and Vural et al. (57). These studies compared several materials used for deep caries management, including Biodentine, calcium hydroxide (CaOH), glass ionomer cement (GIC), calcium-silicate cements (CSC), mineral trioxide aggregate (MTA), and laser-assisted techniques. At 6 months after using an Interprofessional Collaboration (IPC) approach, a meta-analysis was conducted to examine the overall success rate of the materials used. The pooled success rate for IPC was 94.4% with a 95% confidence interval of 89.6–99.2 (Figure 13A). A meta-regression analysis indicated that there was no significant relationship between type of biomaterial used and the success of the treatment when the individual studies were compared. Therefore, all types of biomaterials had comparable short-term outcomes for treatment. The clinical success rate of each of the included studies with calcium-silicate–based materials and conventional liners was high (Figure 13B). At 1 year, the pooled success rate of all treatments remained at 94.4% with a 95% confidence interval of 89.6–99.2 (Figure 14A). A network meta-analysis comparing how effective several types of biomaterials are [Biodentine, CaOH, calcium-silicate cement (CSC), glass ionomer cements (GICs), and laser-assisted techniques] demonstrated that no statistically significant differences were detected between biomaterials; however, overlapping credible intervals and moderate certainty indicate that the evidence is insufficient to establish superiority between interventions (Figure 14B). The ranking probability analysis indicated that calcium-silicate biomaterials as a class, and particularly Biodentine and MTA-based biomaterials within that class, had a higher ranking probability than any other bio-material used for endodontic therapy. It should be noted that several comparisons within this network were informed by sparse data or single-study nodes, which limits the robustness of these estimates. As a result, ranking probabilities should be interpreted cautiously, as they may not reliably reflect true comparative effectiveness (Figure 14C). At the 2-year follow-up, the pooled success rate was 96.0% (95% CI = 91.0–100), though since there were only several studies that reported data at 2-year follow-up, it made it impossible to do a rigorous comparative analysis between these studies (Figure 15). The totality of the evidence supports the strong success rate of tooth preservation with indirect pulp capping for mature teeth, regardless of the particular material being used. There is no statistically significant difference in success rates between classical indirect pulp capping materials [i.e., Ca(OH)2 and glass ionomer cement] and newer calcium silicate-based materials (e.g., biodentine).

### 3.8 Overall success and integrated interpretation

Across all VPT modalities, pooled success rates were consistently high, exceeding 90% at most timepoints, confirming that vital pulp therapy in mature permanent teeth is a reliable treatment approach when appropriate protocols are followed (Table 4).

**Table 4:** Summary of absolute success rates, comparative effectiveness, and certainty of evidence across vital pulp therapy (VPT) modalities in mature permanent teeth.

Modality	Comparison/material	Timepoint	Absolute success (%)	95% CI	Effect direction	Certainty of evidence	Interpretation
Full pulpotomy (FP)	All materials (pooled)	6 months	96.7	92.2–100	—	Moderate	Very high overall success
	All materials (pooled)	1 year	90.8	84.4–97.2	—	Moderate	Sustained high success
	MTA vs. Biodentine	1 year	—	CrIs overlap	No clear difference	Moderate	Inconclusive due to imprecision
	CEM vs. MTA	1 year	—	CrIs overlap	No clear difference	Moderate	Inconclusive
	Biodentine vs CEM	1 year	—	CrIs overlap	No clear difference	Moderate	Comparisons imprecise
	Ranking (all materials)	—	—	—	Biodentine trends higher	Low–Moderate	Ranking uncertain due to sparse network and overlapping estimates
Partial pulpotomy (PP)	All materials (pooled)	6 months	84.0	73.2–94.8	—	Moderate	Moderate success with variability
	All materials (pooled)	1 year	84.8	67.0–100	—	Low–Moderate	Wide uncertainty due to imprecision
	All materials (pooled)	2 years	85.9	70.8–100	—	Moderate	Stable but imprecise estimates
	MTA vs. Biodentine	2 years	—	−0.142 to 0.149	No difference	Moderate	No material-dependent effect detected
	MTA vs. Ca(OH)_2_	6–12 months	—	CrIs overlap	No clear difference	Low	Inconclusive comparison
	Ranking (all materials)	—	—	—	MTA trends higher	Low–Moderate	Ranking uncertain due to wide CrIs
Direct Pulp Capping (DPC)	All materials (pooled)	6 months	89.0	85.0–93.1	—	Moderate	High short-term success
	All materials (pooled)	1 year	89.2	84.5–93.8	—	Moderate	Stable outcomes
	All materials (pooled)	3 years	78.1	70.2–86.1	—	Moderate	Decline over time
	MTA vs. Ca(OH)_2_	6–36 months	—	CrIs exclude 0	Favors MTA	High	Consistent superiority of MTA supported by high-certainty evidence
	Biodentine vs. Ca(OH)_2_	6–36 months		Borderline/narrow CrIs	Favors Biodentine	Moderate	Likely superiority
	MTA vs. Biodentine	All timepoints	—	CrIs overlap	No clear difference	Moderate	Cannot establish superiority
	Ranking (all materials)	—	—	—	Laser/CEM trend higher	Low	Not reliable due to sparse data
Indirect pulp capping (IPC)	All materials (pooled)	6 months	93.2	88.8–97.6	—	Moderate	High short-term success
	All materials (pooled)	1 year	96.8	94.8–98.8	—	Moderate	Very high success
	All materials (pooled)	2 years	98.7	96.1–100	—	Moderate	Sustained high success
	MTA vs Ca(OH)₂	6–12 months	—	CrIs overlap	No clear difference	Moderate	No material advantage demonstrated
	Biodentine vs. Ca(OH)_2_	6–12 months	—	CrIs overlap	No clear difference	Moderate	Inconclusive comparison
	Biodentine vs. MTA	6–12 months	—	CrIs overlap	No clear difference	Moderate	Cannot establish superiority
	Laser vs. other materials	6 months	—	CrIs exclude 0	Less favorable	Low	Sparse and uncertain evidence
	CEM vs. other materials	6 months	—	CrIs variable/sparse	Trends favorable	Low	Based on limited evidence only
	Ranking (all materials)	—	—	—	CEM or MTA trend higher	Low–Moderate	Ranking uncertain due to sparse nodes and overlapping estimates

The degree of certainty associated with comparative effectiveness is a function of the level of certainty associated with the evidence base. Using currently acknowledged data, MTA has been demonstrated to have a higher level of clinical effectiveness compared to calcium hydroxide especially regarding direct pulp capping, and the degree of confidence in this determination is high. Biodentine has demonstrated a better prognosis compared with calcium hydroxide; however, there is a moderate degree of confidence associated with this comparison due to the fact that the prognosis for both materials has wide confidence intervals associated with them.

When compared to each other, the calcium-silicate (MTA and Biodentine) materials yielded overlapping credible intervals demonstrating moderate-level confidence that existing evidence does not support making an absolute claim regarding which material is better than or equal to the other.

Adjunctive therapies (platelet concentrates and laser-assisted techniques) have promising prognoses but are classified by the use of low-grade evidence; thus, the results must be interpreted with caution.

In conclusion, although there is a high degree of evidence indicating that calcium silicate materials have high clinical success and are superior to calcium hydroxide with a high degree of confidence in key comparisons, the relationship of calcium silicate materials to each other remains unclear. Clinical decision-making should therefore integrate both the certainty of available evidence and procedural factors such as asepsis, hemostasis, and quality of coronal seal. It is important to note that absence of statistical significance should not be interpreted as evidence of equivalence, particularly in the presence of wide credible intervals and moderate certainty of evidence.

## 4 Discussion

Across 35 randomized trials (2,906 teeth), vital pulp therapy (VPT) performed with hydraulic calcium-silicate cements—principally mineral trioxide aggregate (MTA), MTA-like premixed HCSCs, and Biodentine—achieved consistently high composite success in mature permanent teeth with carious exposures. This trend continued for full pulpotomy (FP), partial pulpotomy (PP), direct pulp capping (DPC), and indirect pulp capping (IPC) at 6–24 months, with some datasets extending to 36 months. In contrast, calcium hydroxide [Ca(OH)_2_] had lower and less durable outcomes in DPC and PP trials. There were too few studies to justify network density, although the Bayesian and frequentist models converged it was still reasonable to assume the superiority of bioceramics to Ca(OH)_2_, which remained consistent across sensitivity analysis, moderator, and risk-of-bias stratification.

Interpretation of findings was guided by certainty of evidence as assessed using CINeMA. Comparisons supported by high-certainty evidence, such as the superiority of calcium silicate-based materials (MTA, Biodentine) over calcium hydroxide, were considered robust and clinically reliable. In contrast, comparisons among calcium silicate materials themselves were characterized by overlapping credible intervals and moderate certainty, precluding definitive claims of equivalence. Similarly, adjunctive interventions such as laser-assisted therapy and platelet concentrates were supported by low-certainty evidence due to network sparsity and should be interpreted as exploratory rather than confirmatory.

These findings substantiate a paradigm shift for symptomatic adult teeth that historically defaulted to root canal therapy (RCT). In carefully selected cases—especially where asepsis, hemostasis, and immediate coronal sealing are achieved—VPT with modern bioceramics can deliver outcomes that are clinically competitive with more invasive strategies, even in presentations historically labeled as “irreversible pulpitis” (35). Mechanistically, calcium-silicate cements provide alkaline bioactivity, calcium-phosphate interfacial precipitation, and tight sealing, which together support inflammation resolution, tertiary dentinogenesis, and bacterial exclusion (70, 71). The clinical corollary is straightforward: when the clinician has secured rubber-dam isolation, controlled bleeding (e.g., NaOCl), and can place a definitive restoration without delay, bioceramics are the default (72).

These results should be interpreted within the larger context of biologically based endodontics and regenerative endodontics. There are many similarities between vital pulp therapy and revitalization therapies in terms of viable pulp tissue, promoting dentinogenesis, and retaining the dentine-pulp complex. Additionally, numerous studies have shown that regenerative therapies have been demonstrated to effectively treat apical periodontitis by utilizing biological healing processes. As a result, there has been a major shift towards tissue preserving treatments within the field of endodontics. Therefore, vital pulp therapy should be considered not just as another treatment option; rather, it should be viewed as part of a continuum of regenerative therapies used to help maintain or regain the vitality of the pulp based on your patient's diagnosis.

A second, practice-critical point is the unit of success: VPT success is not material alone. Outcomes depend on the biological system comprising (i) diagnosis and case selection, (ii) asepsis and hemostasis, (iii) the pulp-material interface, and (iv) the restoration interface (32). Our analyses repeatedly suggest that immediate, high-quality coronal sealing is at least as decisive as the specific brand of silicate used, especially beyond the early months. This emphasis helps explain why networks with better reporting of isolation/hemostasis and standardized restoration show both higher absolute success and narrower prediction intervals (PIs).

Earlier systematic reviews and meta-analyses established a general advantage of MTA over Ca(OH)_2_ and suggested Biodentine performs similarly to MTA (73–75). The present review advances the field in three ways: Modality-specific networks at clinical timepoints. Rather than pooling modalities (which can obscure transitivity), we analyzed separate networks for FP, PP, DPC, and IPC at 6, 12, 24 (±36) months. This preserves clinical nuance—clinicians do not choose between “VPT in general,” they choose between DPC vs. PP vs. FP in a particular tooth. Prespecified moderators with biological plausibility. We modeled diagnosis (with emphasis on adult symptomatic cases), apex maturity, tooth type, hemostasis protocol, isolation, and restoration strategy. The clearest material signal appeared in DPC, where Ca(OH)_2_ underperformed relative to MTA/Biodentine and material explained a sizable share of between-study variance—consistent with the mechanistic expectation that the earliest interface (exposed pulp) is most sensitive to material properties. Decision-grade outputs. We report relative and absolute effects with PIs and graded certainty for decision-critical contrasts using a network-appropriate framework. This tempers over-reliance on numerical ranks when credible intervals (CrIs) overlap or when single-study nodes (e.g., laser, PRP) inflate apparent performance.

Absolute success is very high across materials. Bioceramics (MTA/Biodentine, plus some PRF-assisted protocols) trend above Ca(OH)_2_ at early and mid-term follow-up, and durability to 24 months is common. In symptomatic adults, Full pulpotomy (FP) is increasingly supported by randomized evidence (35), aligning with our pooled effects and PIs. Pooled success is typically ≥94% at 12 months. Laser-assisted Partial pulpotomy (PP) sometimes rises to the top of rankograms but is usually supported by sparse nodes; we treat such signals as hypothesis-generating. Across better-connected comparisons, MTA vs. Biodentine differences are small and imprecise, whereas Ca(OH)₂ tends to fall lower (36). This is where material choice matters most. At 6 months, Ca(OH)_2_ is significantly inferior to bioceramics in several networks; the gap often persists at longer horizons where data exist (33, 46, 49). Earlier mechanistic/clinical summaries support superior sealing and dentin bridge quality with silicates over Ca(OH)_2_ (76). Clinically, where a Direct pulp capping (DPC) is indicated, bioceramic cements should be first-line. Absolute success is high and stable across materials. MTA-like and Biodentine often place above Ca(OH)_2_ and GIC/RMGIC; however, separations are small and CrIs overlap in several contrasts. Our PIs indicate both MTA-like and Biodentine are reasonable defaults in well-isolated Indirect pulp capping (IPC) when an immediate bonded restoration is planned.

In symptomatic adult cases (SIP), our networks—supported by recent RCTs—challenge the reflex to proceed straight to RCT: full or partial pulpotomy with modern bioceramics can deliver >90%–95% success at 12–24 months when asepsis and hemostasis are rigorously maintained (35), reframing chairside decisions toward a conservative, pulp-preserving option with clear counselling on durability and recall. While immature apices have inherently favourable biology, our synthesis centers on mature permanent teeth (or stratifies mixed cohorts) and shows that outcomes depend more on controllable clinical factors—isolation, bleeding control, and coronal sealing—than on apex status alone; apex maturity may modulate but does not negate the advantage of calcium-silicate cements. Crucially, success tracks the restoration interface: outcomes are consistently superior when a definitive restoration is placed immediately, with well-adapted margins under uncompromised rubber-dam isolation—operatively distilled as rubber dam → NaOCl hemostasis → bioceramic → same-visit definitive restoration—minimizing microleakage and protecting the biological gains achieved at the pulp–material interface.

This review features PRISMA-guided study selection with RoB 2 stratification presented via clear, audit-ready visuals; rigorous outcome harmonization using a composite clinical + radiographic success endpoint to limit definitional drift; modality-specific network meta-analyses at clinically meaningful timepoints to avoid ecological bias from cross-modality pooling; *a priori* moderator analysis grounded in biological plausibility (diagnosis, apex maturity, tooth type, hemostasis, isolation, restoration), translating statistics into practice-level guidance; and reporting of both relative and absolute effects with prediction intervals and certainty grading, which curbs over-interpretation of simple ranks (77).

Evidence networks were sparse for some materials (e.g., laser, PRP/PRF, potassium nitrate), reducing precision and inflating rank volatility; inconsistent reporting of key moderators (hemostasis regimen/duration, isolation breaches, restoration timing/material) complicated transitivity and constrained meta-regression power; unit-of-analysis issues (multiple teeth per patient) were rarely handled at source, leaving potential residual clustering; follow-up attrition and uneven time windows limited certainty beyond 24 months for several contrasts; and heterogeneity in diagnostic thresholds for symptomatic cases introduced classification noise that we mitigated with composite outcomes and sensitivity checks but could not fully eliminate.

For a symptomatic adult molar with deep caries and controlled bleeding, shift from automatic extirpation to a biologically conservative plan: perform full or partial pulpotomy under rubber dam using a modern hydraulic calcium-silicate cement (MTA, MTA-like premixed HCSC, or Biodentine), then place a same-visit definitive restoration. In well-controlled settings, realistic success is >90%–95% at 12–24 months, supported by randomized trials showing pulpotomy can match root canal therapy when asepsis, hemostasis, and sealing are optimized Asgary et al. (35), mature permanent teeth; non-inferiority signals for SIP contexts. Clinically, this reframes counseling: patients can consent to a pulp-preserving option with clear expectations on review intervals and warning signs (pain relapse, lingering sensitivity, or new radiographic findings) that would trigger re-evaluation.

The next gains will come from head-to-head randomized trials among leading silicates (modern MTA formulations, Biodentine, premixed HCSC) within a single modality, using standardized hemostasis and same-visit definitive restoration, with ≥36-month follow-up. Trials should adopt transparent moderator reporting (diagnosis granularity including SIP criteria, apex status, tooth type, exact hemostasis reagent/time, isolation breaches, restoration material/timing) and converge on core outcome sets that standardize composite success definitions and radiographic adjudication—thereby improving meta-analytic fidelity. Pragmatic multicenter designs should integrate patient-reported outcomes (pain trajectory, satisfaction), time/cost, and economic evaluations to inform guidelines and payer policy. Finally, mechanistic adjuncts (e.g., laser, platelet-based concentrates) warrant larger, multicenter evaluations: current signals are encouraging but underpowered for durable hierarchy claims.

There are several important limitations to bear in mind when interpreting the results of this study. The first of these is that several of the modality-to-timepoint combinations had sparsity of the nodes in the network, and thus, particularly with the adjunctive modalities like platelet concentrates and laser-assisted therapies, where only one study exists at a given modality-to-timepoint combination, unstable effect estimates and higher ranking probabilities will be observed. The second limitation is that the clinical heterogeneity among the studies could add to uncertainty about the effect of those treatments; in particular, variability in the preoperative diagnosis, variants in the protocols for hemostasis, confusion in the restoration strategies used, and differing radiographic success criteria may all have affected the outcome of these treatments, even after attempts to evaluate the transitivity of the interventions. Lastly, although formal assessments of publications bias (funnel plots and Egger regression) did not indicate a risk of publication bias in this study, the power of the analyses using these methods for the small number of studies in the respective comparisons makes investigation of the potential for selective publication reporting impossible. The above factors therefore support that based on these limitations, caution should be taken in interpreting the ranking probabilities, and that in clinical decision-making; effect size and certainty of evidence should take precedence over ranking probability.

## 5 Conclusion

Materials that are calcium silicate based, such as MTA or Biodentine yield high success rates in vital pulp therapy for mature permanent teeth. High certainty evidence shows they perform better than calcium hydroxide. However, there is a moderate level of certainty associated with comparative studies using calcium silicate materials and therefore the results cannot be interpreted to mean that they are interchangeable. Evidence that supports adjunctive therapies are low certainty but provide encouraging results. When choosing a material clinically, practitioners should rely on high certainty evidence in addition to taking into consideration all of the procedural factors that accompany the procedure including but not limited to, asepsis, hemostasis, and coronal sealing.

### 5.1 Clinical significance

In adult molars with controlled bleeding and appropriate case selection, vital pulp therapy using calcium silicate-based materials demonstrates high success rates (>90%–95% at 12–24 months). Based on this synthesis, when vital pulp therapy is selected as the treatment strategy, calcium silicate materials are more effective than calcium hydroxide. These findings reflect comparisons within vital pulp therapy modalities and should not be interpreted as direct evidence against root canal treatment.

## Data Availability

The original contributions presented in the study are included in the article/Supplementary Material, further inquiries can be directed to the corresponding authors.
