# Supplementary material for: Decision-ready evidence for vital pulp therapy: a network meta-analysis of bioactive materials in mature permanent teeth
Source: Front Dent Med. 2026 Apr 20;7:1780755. doi: 10.3389/fdmed.2026.1780755 (PMC13136117; doi:10.3389/fdmed.2026.1780755)
Supplement: Supplementary file 1 [file Datasheet1.pdf]

Comparative Effectiveness and Success Rates of 11 Pulp Capping Materials in Vital Pulp Therapy of Cariously Exposed Permanent Teeth - A Systematic Review and Network Meta-analysis.

Firas Elmsmari, Reem Abdelsayed, Qamar Albesoumi

Citation

Firas Elmsmari, Reem Abdelsayed, Qamar Albesoumi. Comparative Effectiveness and Success Rates of 11 Pulp Capping Materials in Vital Pulp Therapy of Cariously Exposed Permanent Teeth - A Systematic Review and Network Meta-analysis.. Not yet published.

PREVIEW

REVIEW TITLE AND BASIC DETAILS

Review title

Comparative Effectiveness and Success Rates of 11 Pulp Capping Materials in Vital Pulp Therapy of Cariously Exposed Permanent Teeth - A Systematic Review and Network Meta-analysis.

Condition or domain being studied

Endodontic Procedure; Pulpotomy ; Pulp Cap, Direct, Excluding Final Restoration; Carious Exposure Of Pulp; Vital Pulpotomy; Pulp Cap Tooth - Indirect

Rationale for the review

Vital pulp therapy (VPT) is a conservative treatment that aims to maintain the health and function of the dental pulp following carious exposure in permanent teeth. It is increasingly favored over root canal therapy, especially when the pulp is still vital and capable of healing. A key factor influencing the success of VPT is the choice of pulp capping material. Calcium hydroxide has long been used, but newer materials such as mineral trioxide aggregate (MTA), Biodentine, and other bioceramics have gained popularity due to their improved sealing ability, biocompatibility, and clinical outcomes.

However, there is still no clear consensus on which material provides the highest success rates, and clinical practices vary. This systematic review aims to compare the effectiveness and clinical success of various pulp capping materials used in VPT for cariously exposed permanent teeth. The findings may help guide evidence-based clinical decision-making and improve long-term treatment outcomes.

Review objectives

Does the choice of pulp capping material play a significant role in the success rate of Vital Pulp Therapy in cariously exposed permanent teeth ?

Keywords

Vital pulp therapy; Direct pulp capping; Indirect pulp capping; Full pulpotomy; Partial pulpotomy; Pulpotomy; Mineral trioxide aggregate; Calcium hydroxide; Bioceramic Materials; Biodentine; Calcium Enriched Mixture; PRF; Glass ionomer cement

Country

United Arab Emirates

ELIGIBILITY CRITERIA

Population

Included

- Permanent teeth

- Carious exposure

- Human Participants

Excluded
- Primary teeth

- Traumatic or mechanical exposure

- Animals

Intervention(s) or exposure(s)

Included
Vital pulp therapy

Excluded
Root Canal Treatment

Comparator(s) or control(s)

Included
PICO tags selected: Pulpotomy ; Pulp Cap, Direct, Excluding Final Restoration; Pulp Cap Tooth - Indirect; Pulpotomy - Vital - Partial; Calcium Hydroxide

Study design
Only randomized study types will be included.

Context
Inclusion Criteria

- Randomized clinical trials with the primary aim of comparing different pulp capping materials in vital pulp therapy (VPT).
- Studies involving permanent teeth with carious pulp exposure.
- Studies reporting success of VPT based on clinical and/or radiographic criteria (e.g., absence of symptoms, radiographic healing, continued root development).
- A minimum follow-up period of 6 months.
- A sample size of at least 10 teeth per treatment group.
- A follow-up rate of 80% or higher to ensure adequate retention and reliability of outcomes.

Exclusion Criteria

- Randomized clinical trials where the primary aim is not the comparison of pulp capping materials in VPT.
- Non-randomized clinical studies, including prospective or retrospective cohort studies, case series, or case reports.
- Animal or in vitro studies.
- Studies involving primary teeth.
- Studies where VPT is performed following traumatic pulp exposure.
- Review articles, editorials, or opinion papers.

SIMILAR REVIEWS

Check for similar records already in PROSPERO
PROSPERO identified a number of existing PROSPERO records that were similar to this one (last check made on 16 June 2025). These are shown below along with the reasons given by that the review team for the reviews being different and/or proceeding.

- Efficiency of different bioceramic materials in direct pulp capping of cariously exposed permanent teeth: A systematic review [published 28 July 2024] [CRD42024567200]. The review was acknowledged as **similar** but the authors opted to continue because *the review looks at additional or different outcomes*, *the review will be more up to date*, *the review uses improved methods*
- Treatment outcomes of vital pulp therapy with different pulp capping materials in permanent teeth [published 19 August 2022] [CRD42022352354]. The review was acknowledged as **similar** but the authors opted to continue because *there are differences in intervention or comparator*, *the review looks at additional or different outcomes*, *the review will be more up to date*, *the review uses improved methods*
- Direct pulp capping for cariously exposed pulp of permanent teeth: systematic review and meta-analysis [published 7 July 2020] [CRD42020179136]. The review was judged **not to be similar**

TIMELINE OF THE REVIEW

Date of first submission to PROSPERO
This record has not been submitted.

Review timeline
Start date: 15 June 2025. End date: 15 December 2025.

Date of registration in PROSPERO
This record has not been published.

AVAILABILITY OF FULL PROTOCOL

Availability of full protocol
A full protocol has not been written.

SEARCHING AND SCREENING

Search for unpublished studies
Only published studies will be sought.

Main bibliographic databases that will be searched
The main databases to be searched are *CENTRAL - Cochrane Central Register of Controlled Trials*, *CLIB - The Cochrane Library* and *PubMed*.

Other important or specialist databases that will be searched
ScienceDirect - Elsevier

Search language restrictions
The review will only include studies published in English.

Search date restrictions
There are no search date restrictions.

Other methods of identifying studies
Other studies will be identified by: *contacting authors or experts* and *searching trial or study registers*.

Link to search strategy
A full search strategy is not available.

Selection process
Studies will be screened independently by at least two people (or person/machine combination) with a process to resolve differences.

Other relevant information about searching and screening
None

DATA COLLECTION PROCESS

Data extraction from published articles and reports
Data will be extracted independently by at least two people (or person/machine combination) with a process to resolve differences.

Authors will not be contacted for further information.

Study risk of bias or quality assessment
Risk of bias will be assessed using: *Cochrane RoB-2*

Data will be assessed independently by at least two people (or person/machine combination) with a process to resolve differences.

Additional information will be sought from study investigators if required information is unclear or unavailable in the study publications/reports.

Reporting bias assessment
Risk of bias due to missing results will be assessed

Certainty assessment
Certainty of the evidence will be assessed using risk of bias, inconsistency and Publication bias.

OUTCOMES TO BE ANALYSED

Main outcomes
Across all treatment modalities, our key outcome measures will include success rates, failure rates, and dropouts, recorded at 6 months, 1 year, 2 years, and 3 years.

Additional outcomes
Secondary outcomes will encompass caries classification, diagnosis, specific teeth involved, restoration time and type, hemostasis measures, infection control protocols, and the operator's details.

PLANNED DATA SYNTHESIS

Strategy for data synthesis
A network meta-analysis (NMA) will be conducted to compare the relative effectiveness and success rates of different pulp capping materials used in vital pulp therapy (VPT) of cariously exposed permanent teeth. This method allows for simultaneous comparison of multiple interventions, even when some have not been directly compared within individual randomized controlled trials.

CURRENT REVIEW STAGE

Stage of the review at this submission

Review stage	Started	Completed
Pilot work	✓	
Formal searching/study identification	✓	
Screening search results against inclusion criteria		
Data extraction or receipt of IPD		
Risk of bias/quality assessment		
Data synthesis		

Review status
The review is currently planned or ongoing.

Publication of review results
Results of the review will be published in English.

REVIEW AFFILIATION, FUNDING AND PEER REVIEW

Review team members
Assistant Professor Firas Elmsmari. Ajman University. United Arab Emirates.

No conflict of interest declared.

Dr Reem Abdelsayed. Ajman University. United Arab Emirates.

No conflict of interest declared.

Dr Qamar Albesoumi. Ajman University. United Arab Emirates.

No conflict of interest declared.

Named contact
Dr Reem Abdelsayed (reem.reem@outlook.com). Ajman University. United Arab Emirates.

Review affiliation

Ajman University

Funding source
Review has no funding and no agreed support from an academic institution and is done in authors' own time.

Peer review
There has been no peer review of this planned review.

ADDITIONAL INFORMATION

Review conflict of interest
Declared individual interests are recorded under team member details.. No additional interests are recorded for this review.

Medical Subject Headings
Pulpotomy; Dental Care; Dental Pulp Capping; Dentition, Permanent; Humans; Network Meta-Analysis

PROSPERO version history
No preview available

Disclaimer
The content of this record displays the information provided by the review team. PROSPERO does not peer review registration records or endorse their content.

PROSPERO accepts and posts the information provided in good faith; responsibility for record content rests with the review team. The guarantor for this record has affirmed that the information provided is truthful and that they understand that deliberate provision of inaccurate information may be construed as scientific misconduct.

PROSPERO does not accept any liability for the content provided in this record or for its use. Readers use the information provided in this record at their own risk.

Any enquiries about the record should be referred to the named review contact
